# Genotype-Phenotype Correlation and Functional Insights for Two Monoallelic *TREX1* Missense Variants Affecting the Catalytic Core

**DOI:** 10.3390/genes13071179

**Published:** 2022-06-30

**Authors:** Giulia Amico, Wayne O. Hemphill, Mariasavina Severino, Claudio Moratti, Rosario Pascarella, Marta Bertamino, Flavia Napoli, Stefano Volpi, Francesca Rosamilia, Sara Signa, Fred Perrino, Marialuisa Zedde, Isabella Ceccherini, on behalf of the Gaslini Stroke Study Group

**Affiliations:** 1 Department of Neuroscience, Rehabilitation, Ophthalmology, Genetics, Maternal and Child Health (DINOGMI), University of Genoa, 16132 Genoa, Italy; giuliaamico@gaslini.org; 2 Laboratory of Genetics and Genomics of Rare Diseases, IRCCS Istituto Giannina Gaslini, 16147 Genoa, Italy; isabellaceccherini@gaslini.org; 3 Center for Structural Biology, Department of Biochemistry, Wake Forest School of Medicine, Winston-Salem, NC 27101, USA; 4 Department of Biochemistry, University of Colorado Boulder, Boulder, CO 80303, USA; 5 Howard Hughes Medical Institute, Chevy Chase, MD 20815, USA; 6 Neuroradiology Unit—IRCCS Istituto Giannina Gaslini, 16147 Genoa, Italy; mariasavinaseverino@gaslini.org; 7 Neuroradiology Unit, Azienda Unità Sanitaria Locale—IRCCS di Reggio Emilia, 42122 Reggio Emilia, Italy; claudio.moratti@ausl.re.it (C.M.); rosario.pascarella@ausl.re.it (R.P.); 8 Physical Medicine and Rehabilitation Unit, IRCCS Istituto Giannina Gaslini, 16147 Genoa, Italy; martabertamino@gaslini.org; 9 Departments of Pediatrics, IRCCS Istituto Giannina Gaslini, 16147 Genoa, Italy; flavianapoli@gaslini.org; 10 Autoinflammatory Diseases and Immunodeficiencies Center, IRCCS Istituto Giannina Gaslini, 16147 Genoa, Italy; stefanovolpi@gaslini.org (S.V.); sarasigna@gaslini.org (S.S.); 11 Biostatistic Unit, Health Science Department (DISSAL), University of Genoa, 16132 Genoa, Italy; francesca.rosamilia@edu.unige.it; 12 Neurology Unit, Stroke Unit, Azienda Unità Sanitaria Locale—IRCCS di Reggio Emilia, 42122 Reggio Emilia, Italy; marialuisa.zedde@ausl.re.it

**Keywords:** TREX1 disorders, CADASIL-like genetics, TREX1 functional assay, stroke genetics, monogenic stroke, small vessel disease, Aicardi-Goutières Syndrome

## Abstract

The TREX1 exonuclease degrades DNA to prevent aberrant nucleic-acid sensing through the cGAS-STING pathway, and dominant Aicardi–Goutières Syndrome type 1 (AGS1) represents one of numerous *TREX1*-related autoimmune diseases. Monoallelic *TREX1* mutations were identified in patients showing early-onset cerebrovascular disease, ascribable to small vessel disease, and CADASIL-like neuroimaging. We report the clinical-neuroradiological features of two patients with AGS-like (Patient A) and CADASIL-like (Patient B) phenotypes carrying the heterozygous p.A136V and p.R174G *TREX1* variants, respectively. Genetic findings, obtained by a customized panel including 183 genes associated with monogenic stroke, were combined with interferon signature testing and biochemical assays to determine the mutations’ effects in vitro. Our results for the p.A136V variant are inconsistent with prior biochemistry-pathology correlates for dominant AGS-causing *TREX1* mutants. The p.R174G variant modestly altered exonuclease activity in a manner consistent with perturbation of substrate interaction rather than catalysis, which represents the first robust enzymological data for a *TREX1* variant identified in a CADASIL-like patient. In conclusion, functional analysis allowed us to interpret the impact of *TREX1* variants on patients’ phenotypes. While the p.A136V variant is unlikely to be causative for AGS in Patient A, Patient B’s phenotype is potentially related to the p.R174G variant. Therefore, further functional investigations of *TREX1* variants found in CADASIL-like patients are warranted to determine any causal link and interrogate the molecular disease mechanism(s).

## 1. Introduction

The Three-prime Repair EXonuclease 1 (*TREX1*) gene (chromosome 3p21.31) encodes a 3′ → 5′ exonuclease, which is ubiquitously expressed in mammalian cells and degrades single-stranded (ss) and double-stranded (ds) DNA substrates in vitro [1,2,3,4]. Structurally, the enzyme is a homodimer of identical 314 amino-acid polypeptides. Each of TREX1′s composing protomers contains an N-terminal catalytic domain (aa. 1–242) and C-terminal tail region (aa. 243–314) [5]. The first is responsible for the protein’s exonuclease activity [2], while the latter facilitates the enzyme’s perinuclear localization [6]. The tail region has also been proposed to interact with the oligosaccharyltransferase (OST) complex [7,8], and TREX1 purportedly interacts with the ER-associated SET complex for DNA repair [9]. 

Early investigations established that *TREX1* knockout mice develop a harsh autoimmune phenotype characterized by inflammatory myocarditis [10], and subsequent functional studies have elucidated that TREX1 functions in vivo to degrade DNA and prevent aberrant DNA-sensing through the cGAS-STING pathway [11,12]. Studies by Perrino et al. [13] and others [14] demonstrate that TREX1′s activity in hematopoietic cells is the deciding factor in whether or not discernable pathology occurs, and the cGAS-STING pathway’s specificity for dsDNA [15,16,17,18,19] suggests TREX1′s pathologically relevant biological substrate is a dsDNA species. To date, multiple biological substrate(s) for TREX1 have been proposed, including DNA replication intermediates [20], retro-elements [21], enucleated erythroblast DNA [22], and micronuclei [23], though this remains an area of active investigation. 

Excessive activation of the cGAS-STING pathway in patients affected by *TREX1* mutations leads to abnormal secretion of type-I interferon (IFN) and nucleic-acid driven inflammation. Genetic diseases that share this IFN-dependent pathogenic mechanism are defined as type-I interferonopathies [24]. Upregulation of IFN-stimulated genes in peripheral blood (“IFN signature”) is common in these conditions [25].

There are four heritable *TREX1*-related disorders [26]: Aicardi–Goutières Syndrome type 1 (AGS1—OMIM #225750), Familial Chilblain Lupus (FCL—OMIM #610448), Systemic Lupus Erythematosus (SLE—OMIM #152700), and Retinal Vasculopathy with Cerebral Leukodystrophy (RVCL—OMIM #192315). Correlations among specific *TREX1* variants, clinical and neuroradiological phenotypes, and their effect on TREX1′s structure/activity have been established. Particularly, monoallelic missense mutations affecting the catalytic domain lead to AGS1 or FCL [26]. For these variants, the molecular disease mechanism is negatively dominant, and likely attributable to the mutant enzymes’ ability to competitively inhibit the wild-type enzyme’s activity on dsDNA substrate [27,28,29,30]. Heterozygous frameshift mutations localized to the C-terminal tail region typically cause RVCL [7,26,31], where the molecular disease mechanism is likely subcellular mislocalization of the protein [6]. Moreover, some biallelic frameshift mutations in the tail region result in AGS1 [26]. Recently, early-onset cerebrovascular diseases ascribable to small vessel disease (SVD) and Cerebral Arteriopathy autosomal Dominant with Subcortical Infarcts and Leukoencephalopathy type 1 (CADASIL)-like neuroimaging pattern have been attributed to heterozygous missense or frameshift *TREX1* mutations [32,33]. These variants affect both the catalytic core and tail region [32,34], but functional characterizations of the identified genotypes with an in vitro heterodimers-based strategy [35] have not been performed. The underlying disease mechanism remains consequently unknown. Of note, the association between the *TREX1* gene and such a phenotype, in the absence of positive family history and/or syndromic features, was not confirmed in some studies [34]; the lack of experimental functional assays represents a declared limitation. 

Moreover, neuroradiological pattern has been recently broadened with a more precise characterization in AGS1 pediatric patients including intracerebral large vessel disease features with a Moyamoya-like arteriopathy [36], but it appears still underscored in cases with adult presentation. Collectively, these observations suggest *TREX1*-associated clinical phenotypes are the result of complex interplay between protein structure/activity, genetic background, and environment.

Herein we report the clinic-neuroradiological features of two patients presenting with AGS-like and CADASIL-like phenotypes, who harbored two different monoallelic, likely pathogenic *TREX1* missense variants affecting the catalytic domain (c.407C>T − p.A136V and c.520A>G − p.R174G). To investigate the potential causal link between the genotypes and observed phenotypes, we performed specific, comprehensive biochemical assays evaluating the mutations’ effects on TREX1′s in vitro exonuclease activity. 

## 2. Results

### 2.1. Clinical and Neuroradiological Features of Patients

#### 2.1.1. Patient A

**Figure 1:**
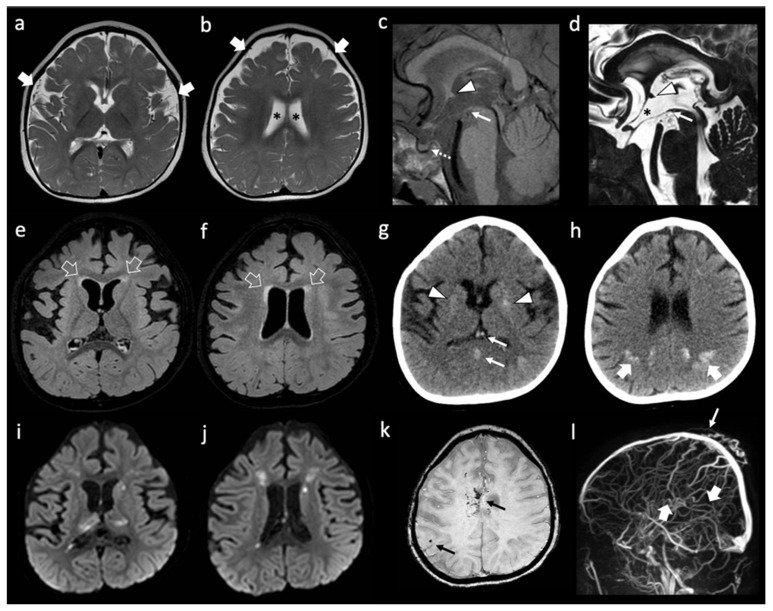
**Neuroimaging features of patient A.** (**a**,**b**) Brain MRI with axial T2-weighted images performed at 1 year of life showing enlargement of the frontal and temporal subarachnoid spaces (thick white arrows) and of the lateral ventricles (black asterisks). Note, the simplified gyration of the cortex, especially in the fronto-temporal lobes. (**c**–**f**) brain MRI performed at 3 years of age with sagittal T1-weighted (**c**) sagittal DRIVE (**d**) and axial FLAIR (**e**,**f**) images reveal dysmorphic anterior commissure (white arrowheads), thin anterior recesses of the third ventricle (black asterisk), absent/dysmorphic mamillary bodies (white arrows), and absent posterior pituitary bright spot (dotted white arrow). There is mild FLAIR hyperintensity of the frontal periventricular white matter (empty white arrows). (**g**,**h**) non-enhanced head CT images performed a few hours after the onset of right hemiparesis, four days after the previous brain MRI, reveal spontaneous hyper-densities of the internal cerebral veins and straight sinus (thin white arrows). In addition, there are subtle, widespread calcifications of the basal ganglia (white arrowheads) and well-demarcated, band-like calcifications of the subcortical parietal white matter (thick white arrows). (**i**–**l**) A brain MRI performed the same day of the CT, including diffusion-weighted images (**i**,**j**) susceptibility weighted imaging (**k**) and phase contrast MR venography (**l**) confirm the absence of blood flow in the straight sinus and internal cerebral veins (thick white arrows) with associated multiple venous ischemic lesions in the thalami and periventricular white matter. In addition, small bleedings are observed in the fronto-mesial and right frontal subcortical white matter (thin black arrows). Note, the parietal median sinus pericranii (thin white arrow).

She is a Caucasian girl who came to our attention at the age of 2 years and 7 months for clinical suspicion of Rapid-onset Obesity with Hypothalamic Dysfunction, Hypoventilation, and Autonomic Dysregulation (ROHHAD) Syndrome [37]. She presented with a severe psychomotor delay, no autonomous de-ambulation, language deficit, axial hypotonia, worsening obesity, episodic fevers in the absence of infection (sterile pyrexias) (T = 39 °C), sleep disorder, constipation, onychodystrophy, and acanthosis nigricans. No other systemic manifestations were present. Blood tests confirmed hypothyroidism, hypo-adrenalism, hypernatremia, hyperchylomicronemia, hypertriglyceridemia, hypercholesterolemia, and diabetes insipidus. Dysmorphic features included plagiocephaly, protruding occipital lumps, thin eyebrows, long pointed chin, and aquiline nose. Cardiological assessment, including ECG, was normal. Brain MRI performed at 1 year of age showed reduced white matter volume with enlargement of the frontal and temporal subarachnoid spaces and simplified gyral pattern (Figure 1a,b). In addition, absence of the posterior pituitary bright spot was noted. A follow-up brain MRI performed at 3 years of age revealed subtle signal alterations of the periventricular white matter in the frontal regions on T2/FLAIR weighted sequences, with stable enlargement of the subarachnoid spaces and lateral ventricles, associated with a parietal median sinus pericranii. Minor dysmorphisms of the third ventricle region were noted, including the abnormal shape of the anterior commissure, the thin, third-ventricle anterior recess, and small/dysmorphic mamillary bodies (Figure 1c–f). After four days, following an episode of dehydration due to acute gastroenteritis, sudden onset of mild right hemiparesis was noted by the parents, and a head CT and brain MRI were performed showing deep cerebral venous thrombosis involving sinus rectus, internal cerebral veins, anterior septal veins, the distal portion of the left basal vein of Rosenthal, and the choroid plexus draining veins (Figure 1g–l). Multiple venous ischemic lesions were evident in the thalami, left striatum, and in the anterior and posterior periventricular white matter (Figure 1i,j). Widespread subtle calcifications of the basal ganglia associated with marked band-like calcifications in the subcortical white matter of the left frontal and bilateral parietal regions were also evident (Figure 1g,h). Continuous intravenous unfractionated heparin (20 U/Kg/h) was administered, then shifted to subcutaneous low molecular weight heparin, and subsequently changed to warfarin. The patient’s family history was positive for cerebrovascular events in the maternal branch. Karyotype, array-CGH, and Sanger sequencing of PHOX2B, MECP2, and FOXG1 genes, as well as a Prader Willi Syndrome methylation test, were negative. Head CT of the father did not show silent cerebrovascular events nor pathological brain calcifications.

#### 2.1.2. Patient B

**Figure 2:**
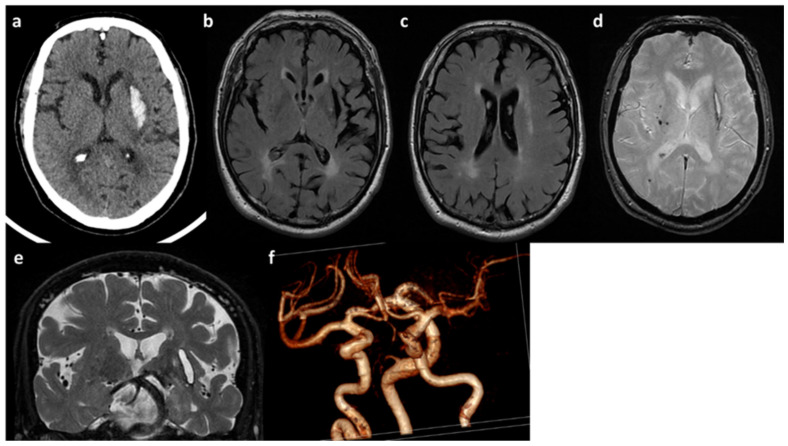
**Neuroimaging features of patient B.** (**a**) Brain CT with acute left ICH in the external capsule. (**b**,**c**) Axial FLAIR sequences of brain MRI with leukoaraiosis and cortical atrophy. (**d**) T2* sequence of brain MRI with lobar microbleeds. (**e**) Coronal T2W sequence of brain MRI with enlarged perivascular spaces and dolichoectatic basilar artery. (**f**) MRA (3D reconstruction) showing the main intracranial arteries with dilatative arteriopathy.

He is a Caucasian male who came to our attention at the age of 55 years because of a deep supratentorial intracerebral hemorrhage (Figure 2a) presenting with dysarthria and right hemiparesis. The past clinical history was unremarkable except for known vascular risk factors: arterial hypertension (upon medical treatment) with mild left-ventricular hypertrophy on transthoracic echocardiography, and obesity (BMI = 30). A further admission in the emergency department was registered at the age of 58 years for amaurosis fugax in the right eye. At that time, he underwent a brain MRI showing a severe SVD pattern [38] with confluent supratentorial leukoaraiosis in the bilateral parieto-occipital white matter (Figure 2b,c), chronic, silent brain infarctions with cavitate appearance involving both the superficial and deep white matter, macro-hemorrhagic scar in the left external capsule and several infratentorial and supratentorial microbleeds (Figure 2d), increased enlarged perivascular spaces mainly in the deep supratentorial compartment (Figure 2e), and significant cortical atrophy at the visual rating. Intracranial MRA sequence (Figure 2f) was significant for severe intracranial dilatative arteriopathy with prominent vertebral-basilar dolichoectasia. No systemic manifestations were present. 

### 2.2. Genetic Analysis Findings 

The results of the genetic analysis are summarized in Table 1. The monoallelic likely pathogenic c.407C > T (p.A136V) and c.520A > G (p.R174G) *TREX1* variants in patient A and B, respectively, were identified (Supplemental Figure S1). Segregation study in patient A confirmed a paternal inheritance (data not shown), whereas it was not possible to study patient B’s family, since he was adopted and his biological parents are unknown. 

No other deleterious variants were found in the genes included in the genetic panel. The variants of uncertain significance, listed in Table 1, were determined to be inconsistent with the neuroradiological and clinical phenotypes. 

### 2.3. Peripheral Blood Type I Interferon Activity

**Figure 3:**
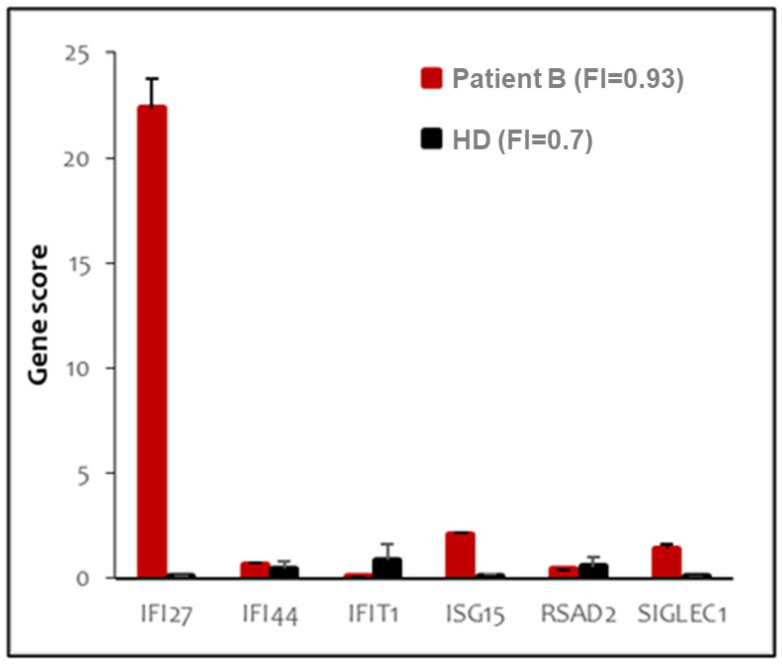
**Peripheral blood type I interferon signature of patient B.** The panel shows the expression of six type I-IFN stimulated genes, indicated in the *X* axis. Red bars indicate expression levels in patient B; black bars represent the average of twenty healthy donors. Interferon score (FI) is indicated in the legend.

In order to gain insight into a possible inflammatory mechanism underlying the patients’ phenotypes, we determined their peripheral blood type-I IFN score (IS), which reliably tests positive in AGS1 patients [25]. We identified a negative IS for patient A (data not shown) and moderately positive IS for patient B (Figure 3) (IS = 0.93, normal values < 0.7). We note that certain gene scores have high leverage (IFI27) in the overall IS, whereas others showed more minor (ISG15, SIGLEC1) or negligible (IFI44, IFIT1, RSAD2) change. 

### 2.4. Exonuclease Activities of Mutant Enzymes

**Figure 4:**
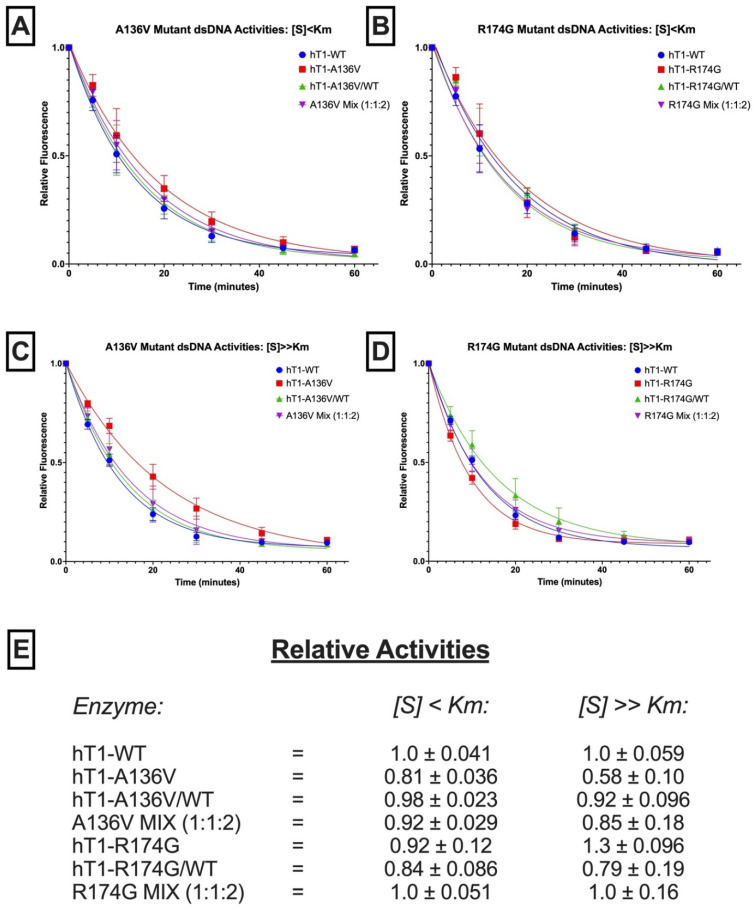
**dsDNA exonuclease activities of p.A136V and p.R174G variants.** (**A**–**D**) *Fluorescence-Based Quantification of TREX1 dsDNA Degradation*. Standard exonuclease reactions were prepared with equimolar concentrations of the indicated enzymes/mixes, incubated at room temperature for the indicated times, quenched in SYBR Green, and dsDNA content measured by fluorescence. Plots of fluorescence vs. time were generated and fit with one-phase decay nonlinear regression in Prism 7.0 (GraphPad). Plots are composites of 18 different reactions from three different experiments. Data points indicate mean, and error bars represent standard deviation. (**E**) *Activity Rates of TREX1 Variants*. Initial velocities were quantified from the respective regression lines in panels a-d and normalized to wild-type initial velocity to calculate relative activity. Values are mean and standard deviation. ‘[S] < K_m_’ refers to ~1 nM of a 10-kb dsDNA plasmid, and ‘[S] >> K_m_’ refers to ~1 µM of a 30-bp self-annealing oligo.

**Figure 5:**
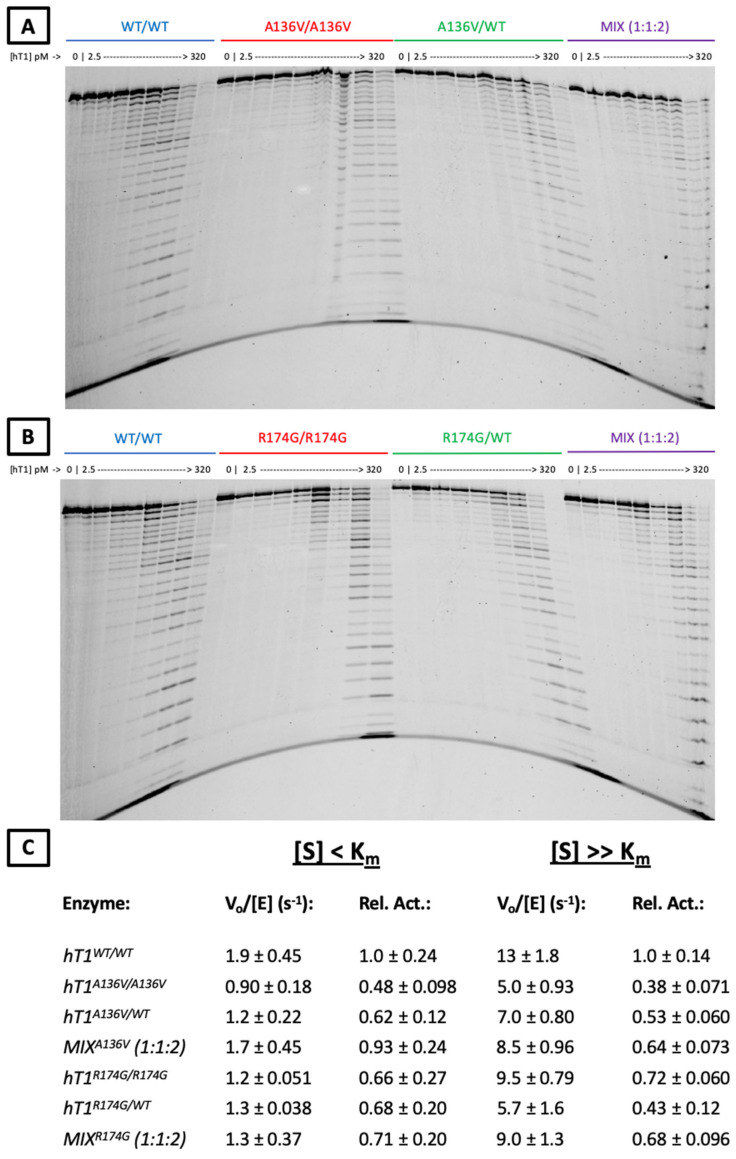
**ssDNA exonuclease activities of p.A136V and p.R174G variants.** (**A**,**B**) *Urea-Polyacrylamide Gel Visualization of TREX1 Exonuclease Activity*. Standard exonuclease reactions were prepared with indicated concentrations of the listed enzymes, incubated at room temperature for 20 min, quenched in ethanol, then visualized on a 23% urea-polyacrylamide gel. Top bands indicate undegraded 30-mer oligonucleotide, which TREX1 degrades in single-nucleotide units to generate the laddered bands. (**C**) *Activity Rates of TREX1 Variants*. Reactions such as those in panels a-b were carried out with nine separate reactions per enzyme across three different enzyme concentrations. Densitometric quantification of these gels was used to calculate activity rates, which were converted to relative activities by normalization to the wild-type activity rate. Values are mean and standard deviation. ‘[S] < K_m_’ refers to ~1 nM of a 10-kb dsDNA plasmid, and ‘[S] >> K_m_’ refers to ~1 µM of a 30-bp self-annealing oligo.

The affected residues are situated in distinct regions of the TREX1 catalytic core (Supplemental Figure S2), which is entirely responsible for the enzyme’s exonuclease activity and dispensable for normal localization [2,6]. R174 is located on an active-site adjacent flexible loop, which analogous TREX2 studies suggest participates in DNA binding [39,40], whilst A136 resides within TREX1′s α6 helix is farther from the active-site and partially buried within the protein’s surface. Previous interrogation of the effect of the p.R174A substitution on TREX1 exonuclease activity in vitro found a modest deficit in dsDNA exonuclease activity [30], but no prior biochemical data regarding A136 mutations exist in the literature. We overexpressed and purified all dimeric variants of recombinant human TREX1_1–242_ (hT1) presumably present in the aforementioned patients (hT1^+/+^, hT1^A136V/+^, hT1^A136V/A136V^, hT1^R174G/+^, and hT1^R174G/R174G^). Then, we quantified their ss- and dsDNA exonuclease activities (Figure 4 and Figure 5) with validation by alternative assays (Supplemental Figures S3 and S4), and we utilized concentrations of free 3′-hydroxyls (TREX1 substrate concentration) above ([S] >> Km) and below ([S] < Km) TREX1′s reported K_m_ values [2]. Since sufficiently high, substrate concentrations (enzyme-limiting) should lead to steady-state occupation of essentially all active-sites, deficits to substrate binding should have differential effects on enzymatic activity dependent on the substrate concentration, while deficits to catalysis should affect enzymatic activity at all substrate concentrations. Thus, our assay can distinguish the mutations’ effects on TREX1′s substrate binding versus catalysis.

Regarding p.A136V, hT1^A136V/+^ and hT1^A136V/A136V^ did not exhibit compelling differences in dsDNA exonuclease activity versus wild-type enzyme (Figure 4E) once variation between the protein preps was accounted for (Supplemental Figure S5), but both displayed ~2-fold reduced relative ssDNA exonuclease activity under all tested substrate concentrations (Figure 5C). Moreover, a genotype mix of the p.A136V mutant enzymes (1:1:2 ratio of hT1^+/+^:hT1^A136V/A136V^:hT1^A136V/+^) exhibited comparable exonuclease activity to wild-type enzyme for all substrates and concentrations, except for a minor (<2-fold) decrease in relative activity with above-K_m_ concentrations of ssDNA substrate. 

Regarding p.R174G, none of the variants nor their genotype mix (1:1:2 ratio of hT1^+/+^:hT1^R174G/R174G^:hT1^R174G/+^) had a significant effect on relative dsDNA exonuclease activity, except for a modest (<2-fold) increase in relative exonuclease activity for hT1^R174G/R174G^ on the above-K_m_ dsDNA substrate (Figure 4E). All tested p.R174G mutants displayed a modest (≤2-fold) decrease in relative ssDNA exonuclease activity that was not significantly impacted by substrate concentration (Figure 5C). Finally, comparison of the observed (α) versus theoretical (α^*^) exonuclease activities of the p.A136V (ssDNA_[S] < Km_: α = 0.93 ± 0.24, α^*^ = 0.68 ± 0.088; ssDNA_[S] >> Km_: α = 0.64 ± 0.073, α^*^ = 0.61 ± 0.049; dsDNA_[S] < Km_: α = 0.92 ± 0.029, α^*^ = 0.94 ± 0.018; dsDNA_[S] >> Km_: α = 0.85 ± 0.18, α^*^ = 0.86 ± 0.056) and p.R174G (ssDNA_[S] < Km_: α = 0.71 ± 0.20, α^*^ = 0.76 ± 0.13; ssDNA_[S] >> Km_: α = 0.61 ± 0.096, α^*^ = 0.64 ± 0.071; dsDNA_[S] < Km_: α = 1.0 ± 0.051, α^*^ = 0.90 ± 0.053; dsDNA_[S] >> Km_: α = 1.0 ± 0.16, α^*^ = 0.97 ± 0.10) variants’ genotype mixes do not indicate any gross dominant-negative effects (see ‘Observation-Theory…’ in Methods for more details). 

## 3. Discussion

Although the genotype-phenotype correlation for AGS1 (OMIM #225750), FCL (OMIM #610448), SLE (OMIM #152700), and RVCL (OMIM #192315) has been experimentally established [26,27,28,29,30], the association between *TREX1* genotypes and early-onset cerebrovascular disease, owing to SVD and CADASIL-like neuroradiological pattern, lacks systematic functional characterization via an in vitro heterodimers-based strategy [35]. Moreover, missense variants are in general responsible for a broad range of biological effects resulting in a varied spectrum of phenotypic consequences. For this reason, their interpretation might be challenging. Specific functional applications represent a powerful tool in support of pathogenicity, especially when genotypes, even if predicted in silico as “deleterious”, are associated with disease variability [41]. 

In this study, we report the clinical-neuroradiological features of two patients with AGS-like (Patient A) and CADASIL-like (Patient B) phenotypes who carried the heterozygous likely pathogenic c.407C > T (p.A136V) and c.520A > G (p.R174G) *TREX1* missense variants, respectively. To interrogate a potential causal link between these genotypes and phenotypes, genetic findings were combined with specific, comprehensive biochemical assays assessing TREX1′s in vitro exonuclease activity.

Concerning the p.A136V variant harbored by Patient A, the in vitro evaluation of the related TREX1 dimeric enzyme variants did not reveal any effects consistent with dominant AGS1: the mutant produces a ≤2-fold decrease in ss- and dsDNA exonuclease activity without exhibiting negative biochemical dominance [28,29,30]. Similarly, the familial segregation study confirmed paternal inheritance, but a head-CT of the father tested negative for silent cerebrovascular events and brain pathological calcifications. Also, the type I-IFN signature was normal as in the proband. While minor biochemical perturbations can sometimes lead to significant clinical outcomes, such a relationship is unprecedented for *TREX1* and dominant AGS. All well-characterized dominant AGS-causing *TREX1* mutations produce a mutant enzyme that is catalytically inactive and able to competitively inhibit the activity of the wild-type counterpart [26,29,30,42]. 

In addition, all existing biochemistry-pathology relationship studies for other *TREX1* disease-associated variants (reviewed in ref [26]) indicate that those with residual exonuclease activity (typically biallelic variants with high penetrance) still have a greater than 2-fold deficit as mutant homodimers. These evidences are in stark contrast to our observations for p.A136V. It may be hypothesized that p.A136V could impact other biological mechanisms such as subcellular localization or protein-protein interactions, however the complete dispensability of TREX1′s catalytic core for proper localization [2,6] and A136′s non-surface location (Supplemental Figure S2) make these two specific alternatives unlikely. Overall, while we acknowledge the ever-present complication of in vivo versus in vitro discrepancies, including the presence of other DNA-associating factors on TREX1′s biological substrate, taken together our data suggest the p.A136V variant is not causative for dominant AGS in Patient A. We may not rule out that p.A136V partially contributes to other diseases in other genetic and environmental contexts, nor that Patient A has a complex and poorly understood non-AGS phenotype to which p.A136V partially contributes. 

The effect of the p.R174G variant harbored by Patient B was experimentally investigated for the first time. The type-I IFN signature was minor to moderate, with high variability in gene scores among the IFN signature panel, and this may warrant a more exhaustive future investigation of interferon-stimulated gene expression to robustly characterize IFN signatures for such a phenotype. The family history is unknown since he was adopted, and his currently asymptomatic offspring declined participation in a segregation study. Biochemical interrogations revealed modest perturbations of TREX1 ss- and dsDNA exonuclease activities, with R174G dimeric variants being within 2-fold of wild-type enzyme and the genotype mix displaying normal activity with no indication of negative biochemical dominance [28,29,30]. This magnitude of deficit is comparable to that previously published for the TREX1 R174A variant [30]. Without established *TREX1* biochemistry-pathology corollaries for Patient B’s phenotype, it is difficult to ascertain whether such exonuclease dysfunction is pathologically relevant. In fact, the historical association between *TREX1*-related dominant phenotypes and the dramatic reduction in the exonuclease activity [11,26], together with our data related to the p.A136V mutant, would suggest the p.R174G-mediated enzymatic dysfunction is not sufficient on its own to cause Patient B’s pathology. However, the mutation is located on TREX1′s proposed flexible binding loop, and the dependence of our dimeric variants’ relative exonuclease activities on substrate type and concentration are not consistent with strict catalytic perturbation, implying that the R174 residue directly participates in more nuanced interactions between TREX1 and DNA that make it unique among prior, disease-associated *TREX1* mutations (reviewed in reference [26]). Since substrates in our in vitro studies are unlikely to wholly recapitulate interaction with TREX1′s biological substrate, there remains the possibility for more drastic activity deficits in vivo and/or a causal link between p.R174G and Patient B’s phenotype. Overall, our data suggest a potential association between the *TREX1* p.R174G mutation and a dominant CADASIL-like phenotype, which warrants further investigation. 

In conclusion, our multidisciplinary evaluation including neuroradiological, clinical, and genetic investigations combined with functional biochemical assessments of the identified genotypes led to a global interpretation of Patients A and B’s phenotypes. Patient A presented with a complex clinical and neuroradiological phenotype, not entirely ascribable to ROHHAD Syndrome but, as demonstrated here, unlikely related to AGS1. The p.A136V variant likely represents a rare *TREX1* polymorphism, with minimal pathological impact on its own. In this case, whole exome sequencing may clarify whether novel candidate genes or other known disease genes, not considered in the applied NGS panel, are responsible for Patient A’s phenotype. For Patient B, a clinical and neuroradiological phenotype was typically suggestive of an SVD with a CADASIL-like pattern. The p.R174G variant was previously identified in affected individuals showing same neuroradiological features in a large cohort [33] and case-control [34] studies. In contrast to other *TREX1* variants, p.R174G was never found in matched healthy controls [34]. Our biochemical investigations identified effects on TREX1 exonuclease activity that were more consistent with perturbation of substrate interaction than catalysis, but the effects’ modest magnitudes are not consistent with any prior, *TREX1*-associated, dominant phenotypes. Despite this, p.R174G’s novel perturbation of TREX1′s substrate binding (as opposed to catalysis or localization), the lack of prior biochemistry-pathology corollaries for *TREX1*-associated SVD with CADASIL-like pattern, the rare allelic frequency, the pathogenicity scores, and the absence of homozygous individuals in all the ethnic groups (Table 1), leave open the possibility of a clinical causality for this variant. This study represents the first robust enzymological data for a *TREX1* variant identified in a CADASIL-like patient: further experimental investigations of *TREX1* variants identified in patients with this neuroradiological phenotype are warranted to elucidate the molecular disease mechanism(s) underlying such pathology. 

## 4. Material & Methods

### 4.1. Genetic Analysis

DNA was isolated from the peripheral blood samples of patients, and parents when available, and extracted by using QIAamp DNA Blood Midi kit (Qiagen). Informed consent was obtained, and pre-test and post-test genetic counselling were provided [43]. A customized genetic panel was designed through the Ion AmpliSeq Designer Software (https://www.ampliseq.com—accessed on 10 July 2019), including 183 genes associated with monogenic forms of cerebral stroke (Supplemental Table S1). Genes were selected according to a deep review of the current literature and the Online Mendelian Inheritance in Man database [44]. Coding regions, plus at least 5bp flanking each exon, were included in the panel’s design for a total target of 4595 amplicons spanning 897.22 kb. The average length of the amplimers was 125–175 base pairs. Library preparation and successive DNA sequencing were performed according to manufacturer’s protocols on PGM^TM^ (Thermo-Fisher) and Ion S5 System supplied with Ion Chef System (Thermo-Fisher). Missed regions, amounting to 1771 bp, and selected amplicons displaying a coverage less than 10X were analysed via Sanger sequencing when necessary. 

### 4.2. Bioinformatic Analysis of NGS-Panels Genetic Data

FastQ data were assessed, and variants were called by the IonReporter^TM^ Software v5.16.02. A custom pipeline was additionally optimized to filter variants based on minor allele frequency (MAF) ≤ 1%, impact on the mature, encoded protein (missense, nonsense, frameshift, splicing variants ±2 bp from the exon boundaries), in silico functional prediction using CADD [45], Polyphen [46] and SIFT [47] tools, and/or pathogenicity classification provided by the Varsome [48] and Clinvar [49] databases, according to the American College of Medical Genetics and Genomics [50,51]. Variants were reported as pathogenic, likely pathogenic, of uncertain significance, likely benign, and benign. Variants of uncertain significance were interpreted by a multidisciplinary team, including clinicians, neuroradiologists, and geneticists to ascertain a possible genotype-phenotype correlation as recommended by The Association for Clinical Genomic Science Best Practice Guidelines for Variant Classification in Rare Disease 2020 (unpublished work). Therefore, selected variants were validated via Sanger sequencing and evaluated according to clinical manifestations in the probands and to parental segregation, whenever possible.

### 4.3. Peripheral Blood Type I Interferon Signature

The IFN signature was performed as described [52] with modifications, measuring six IFN stimulated genes’ (*IFI27*, *IFI44*, *IFIT1*, *ISG15*, *RSAD2*, *SIGLEC1*) expression by real time PCR. A synthetic control was used to determine copy numbers of the selected genes.

The IFN signature for each subject was calculated as the geometric mean of the genes’ copy numbers normalized using two endogenous reference genes (*HPRT1* and *G6PD*). Cut-off value was obtained as the mean + 2SD of 20 healthy donors’ IFN signature. 

### 4.4. Generation of Wild-Type and Mutant TREX1 Plasmids

A pCDFDuet-1 vector containing the wild-type (WT) coding sequence of human *TREX1* gene (NM_033629) (1–242 residues) was amplified and then the c.407C>T − p.A136V and the c.520A>G − p.R174G variants were separately inserted by site-directed mutagenesis. 

### 4.5. Overexpression and Purification of Recombinant TREX1 Enzymes

Purification of TREX1 enzymes was performed using our previously published methodology [35]. Briefly, truncated recombinant human TREX1_1–242_ (hT1) enzymes were produced for WT enzyme (hT1^+/+^) and all the dimeric variants of the mutants presumably present in patient A (hT1^A136V/+^ and hT1^A136V/A136V^) and patient B (hT1^R174G/+^ and hT1^R174G/R174G^). hT1 mutant protomers were expressed as a fusion protein using a pCDF-Duet vector that encodes His-tagged NusA linked N-terminally to hT1. WT protomers were expressed as a fusion protein using a pLM303x vector that encodes maltose-binding protein (MBP) linked N-terminally to hT1. Both constructs employed a linker with a rhinovirus 3C protease (PreScission Protease) recognition site and were transformed into *Escherichia Coli* Rosetta2 (DE3) cells (Novagen) for overexpression. 

For homodimers, only the respective constructs were transformed into cells. WT and mutant homodimers were purified via amylose column chromatography and nickel column chromatography, respectively, followed by overnight PreScission Protease treatment, then phosphocellulose (p-cell) column chromatography. For heterodimers, the WT and relevant mutant construct were co-transformed into cells for co-expression, then purified via sequential amylose (to remove mutant homodimers), nickel (to remove wild-type homodimers), and p-cell column (to remove cleaved MBP/NusA and trace contaminants) chromatography, with overnight PreScission Protease treatment between the nickel and p-cell columns. Amylose and nickel columns used one-step elution, except for the hT1^A136V/A136V^ enzyme purification, which required gradient elution off the nickel column. All p-cell columns used a salt gradient for elution. WT and p.R174G variant eluted from p-cell columns at a salt concentration of ~120 mM, while p.A136V variant eluted at ~200 mM. Enzyme concentrations of preps were determined by A_280_ on a NanoDrop 2000 spectrophotometer (ε = 23,950 M^−1^cm^−1^) and validated by SDS-PAGE (Supplemental Figure S5). 

### 4.6. Fluorescence-Based dsDNA Exonuclease Assay

As previously published [35], 150 µL reactions were prepared containing a variable concentration of hT1, 20 mM TRIS (pH 7.5 @ 25 °C), 5 mM MgCl_2_, 2 mM dithiothreitol (DTT), 5 ng/µL dsDNA substrate, and 200 ng/µL bovine serum albumin (BSA). For the ‘[S] < K_m_’ experiments (see Figure 4), the dsDNA substrate was a ~10-kb Nt.BbvCI-nicked (NEB #R0632) pMYC plasmid used at 5 ng/µL (~0.83 nM), and the hT1 concentration was 7.5 nM. For the ‘[S] >> K_m_’ experiments (see Figure 4), the dsDNA substrate was a self-annealing 30-mer oligo used at 1000 nM (~9 ng/µL), and the hT1 concentration was 1.25 nM. Reactions were initiated by addition of 10X enzyme diluted in 1 mg/mL BSA and incubated for 1 h at room temperature (~21ºC). Then, 20 µL samples were taken at 0, 5, 10, 20, 30, 45, and 60-min time points and quenched in 20 µL of 15X SYBR Green dye (ThermoFisher). Quenched samples had their fluorescence measured on a PolarStar Omega microplate reader (BMG LabTech), using excitation/emission of 485/520 nm. 

The 30-mer oligo sequence: 5′-GCTCGAGTCA TGACGCGTCA TGACTCGAGC-3′

For these experiments, 1:1:2 mix ratios indicate the prevalence of hT1^+/+^, hT1^MUT/MUT^, and hT1^MUT/+^, respectively, where the described concentrations represent the total molarity of all variants in the mixes. Each independent enzyme dilution in each experiment was used to create six replicate reactions. 

### 4.7. Fluorescence-Based ssDNA Exonuclease Assay

To begin, 150 µL reactions were prepared containing a variable concentration of hT1, 20 mM TRIS (pH 7.5 @ 25 °C), 5 mM MgCl_2_, 2 mM dithiothreitol (DTT), 5 µM ssDNA substrate, and 200 ng/µL bovine serum albumin (BSA). The ssDNA substrate was a 30-mer oligo used at 5 µM (~45 ng/µL), and the hT1 concentrations were 3 nM. Reactions were initiated by addition of 10X enzyme diluted in 1 mg/mL BSA, and incubated for 1 h at room temperature (~21ºC). 20 µL samples were taken at 0, 5, 10, 20, 30, 45, and 60-min time points and quenched in 20 µL of 1% QuantiFluor^®^ ssDNA dye (Promega #E3190) plus 10 mM EDTA. Quenched samples had their fluorescence measured on a PolarStar Omega microplate reader (BMG LabTech), using excitation/emission of 485/520 nm.

The 30-mer oligo sequence: 5′-TTAACCTTCT TTATAGCCTT TGAACAAAGG-3′

For each of the five experiments per substrate, eight independent enzyme dilutions were prepared: two hT1^+/+^, one hT1^A136V/A136V^, one hT1^A136V/+^, one p.A136V-variant mix, one hT1^R174G/R174G^, one hT1^R174G/+^, and one p.R174G-variant mix. In these experiments, 1:1:2 mix ratios indicate the prevalence of hT1^+/+^, hT1^MUT/MUT^, and hT1^MUT/+^, respectively, where the described concentrations represent the total molarity of all variants in the mixes. Each independent enzyme dilution in each experiment was used to create six replicate reactions. 

### 4.8. Quantification of Fluorescence-Based Data

For each of the A136V/R174G experiments per substrate, eight independent enzyme dilutions were prepared including two independent wild-type dilutions. Each independent enzyme dilution in each experiment was used to create six replicate reactions. Plots of fluorescence vs. time were generated from the time-course data for every reaction in each experiment; then, each reaction was normalized to initial fluorescence and plate background fluorescence. The normalized data points from every reaction replicate of each independent enzyme dilution were used for fitting with one-phase decay nonlinear regression. This gave eight fitted datasets per experiment, one for each independent enzyme dilution. The slope of each fit at 0 min was interpolated to give initial velocities, and then initial velocities were normalized within each experiment to the mean of the wild-type fits’ initial velocities to calculate relative activities for each variant/mix. The relative activities across the five experiments for each substrate were then used to calculate mean and standard deviation of relative activities. Calculations were performed in Excel, and regression and interpolation were performed in Prism 7.0 (GraphPad).

### 4.9. Agarose Gel dsDNA Assay

As previously published [35], 100 µL reactions were prepared containing 15 or 30 nM hT1, 20 mM TRIS (pH 7.5 @ 25 °C), 5 mM MgCl_2_, 2 mM dithiothreitol (DTT), 10 ng/µL nicked dsDNA substrate, and 100 ng/µL bovine serum albumin (BSA). Substrate was generated by incubation of ~10-kb pMYC plasmid with Nt.BbvCI (NEB #R0632) nicking enzyme, per vendor specifications. Reactions were initiated by addition of 10X enzyme diluted in 1 mg/mL BSA and incubated at room temperature (~21ºC). Then, 20 µL samples were taken at indicated time points and quenched in 80 µL of cold ethanol. Quenched samples were dried in vacuo, resuspended in a loading buffer, then electrophoresed on a 0.8% agarose gel and imaged on a Typhoon FLA 9500 (GE Healthcare Life Sciences). For these experiments, the variant mixes were 1:1:2 ratios of hT1^+/+^, hT1^MUT/MUT^, and hT1^MUT/+^, respectively, where the described concentrations represent the total molarity of all variants in the mixes. 

### 4.10. Quantification of Agarose Gels

Agarose gel images were subjected to 1D densitometry using the GelAnalyzer 2010a software. Lanes were identified automatically with software defaults, and intensity vs. migration functions for each lane were subjected to background subtraction by the rolling ball method with a ‘500’ ball radius setting. Next, the band peak corresponding to the starting substrate (‘Nicked’) was manually identified for the ‘0-minute’ lane of each reaction, then quantified by integration over the migration range of the band. The maximum and minimum migration values of these peaks were noted for each reaction and used to manually define the integration range for signal quantification of the reactions’ other time-points/lanes. Then, the integral values for each reaction were normalized internally to their maximum using Excel. This provided normalized values for each reaction at each time point, which were indicative of the proportion of initial nicked plasmid remaining over time for each reaction. 

### 4.11. Polyacrylamide Gel ssDNA Assay

As previously published [35], 100 µL reactions were prepared containing a variable concentration of hT1, 20 mM TRIS (pH 7.5 @ 25 °C), 5 mM MgCl_2_, 2 mM dithiothreitol (DTT), a variable concentration of ssDNA substrate, and 100 ng/µL bovine serum albumin (BSA). Reactions were initiated by addition of 10X enzyme diluted in 1 mg/mL BSA and incubated for 20 min at room temperature (~21ºC). Reactions were quenched in 400 µL cold ethanol, dried in vacuo, resuspended in loading solution, then electrophoresed on a 23% urea-polyacrylamide sequencing gel and imaged on a Typhoon FLA 9500 (GE Healthcare Life Sciences).

For these experiments, the variant mixes were 1:1:2 ratios of hT1^+/+^, hT1^MUT/MUT^, and hT1^MUT/+^, respectively, where the described concentrations represent the total molarity of all variants in the mixes. For the comparative gels, the substrate was a 5′-FAM-labeled non-annealing 30-mer oligo used at 15 nM, and the hT1 concentrations ranged from 2.5–320 pM in 2-fold increments. For the ‘[S] < K_m_’ quantification experiments, the substrate was a 5′-FAM-labeled non-annealing 30-mer oligo of the above sequence used at 15 nM. For the ‘[S] >> K_m_’ quantification experiments, the substrate was a mix of 5′-FAM-labeled and unlabeled non-annealing 30-mer oligos of the above sequence used at 15 nM and 500 nM, respectively, and the hT1 concentrations ranged from 20–320 pM. For the experiments used for quantification, three different hT1 concentrations were used per variant/mix, and each concentration included three reaction replicates. For the ‘[S] < K_m_’ quantification experiments, wild-type used 5, 10, and 20-pM, and the p.A136V and p.R174G variants/mixes used 10, 20, and 40-pM. For ‘[S] >> K_m_’ quantification experiments, wild-type used 40, 80, and 160-pM, and the p.A136V and p.R174G variants/mixes used 80, 160, and 320-pM. In addition, a single reaction with an increased concentration of wild-type enzyme was used to generate a full ladder of oligo sizes for reference during quantification. 

5′-FAM-labeled 30-mer oligo sequence: 5′-[FAM] ATACGACGGT GACAGTGTTG TCAGACAGGT-3′

### 4.12. Quantification of Polyacrylamide Gels

Agarose gel images were subjected to 1D densitometry using the GelAnalyzer 2010a software. Lanes were identified automatically with software defaults, and intensity vs. migration functions for each lane were subjected to background subtraction by the rolling ball method with a ‘500’ ball radius setting. Next, the peaks in each lane, corresponding to various oligo sizes, were manually identified using the aforementioned internal ladder as a visual reference for the expected migration positions. Then, peaks were integrated automatically to quantify relative oligo levels for all bands. These band values were used to calculate the initial velocities of the reactions, via methods [35] that we have previously detailed. Means and standard deviations of initial velocities were calculated using the values for the nine reactions per enzyme/mix (three replicates each for three enzyme concentrations). Relative activities were calculated by normalization to the mean initial velocities of the wild-type reactions. 

### 4.13. Observation-Theory Comparisons for Exonuclease Activities

Theoretical activities of the 1:1:2 genotype mixes of the mutants’ variants were calculated as independent composites via Equations (1) and (2). Mean relative activities of the respective variant dimers are indicated by *µ*, and standard deviations are indicated by *σ*; values were calculated from all experimental and reactions replicates for the respective primary assays determining relative activity rate.

(1) $$\mu^{MIX}=0.25\;\mu^{+/+}+0.25\;\mu^{MUT/MUT}+0.5\;\mu^{MUT/+}$$

(2) $$\sigma^{MIX}=\;\sqrt{{\left(0.25\;\sigma^{+/+}\right)}^{2}\;+{\left(0.25\;\sigma^{MUT/MUT}\right)}^{2}+{\left(0.5\;\sigma^{MUT/+}\right)}^{2}}$$

### 4.14. Modeling of Human TREX1 Enzyme Structure

The published structure of mTREX1_1–242_ bound to ssDNA (PDB = ‘2IOC’) was modified in PyMOL v2.3.2 [53] to remove nucleic acid and ions. The modified structure was then used as a template for MODELLER v9.22 [54] to convert the murine sequence to human and model in the flexible loops missing from the original structure. The original mTREX1 published structure was then loaded into PyMOL [53] and aligned to the human TREX1 model. The calcium ions and ssDNA polymers from the original structure were superimposed on the human TREX1 model, and the calcium ions converted to magnesium, to generate the model shown in Supplemental Figure S2. 

## Figures and Tables

**Table 1:** Genetic results obtained from the NGS-panel analysis.

pt		Variants Description											
	GENE	cDNA Change	Protein Change	dbSNP	zig	seg	Varsome	Clinvar	CADD	MAF *	Hom ^#^	Polyphen	SIFT
A	TREX1	c.407C>T	p.A136V	rs1560112354	het	pat	LP	-	28	0.8 × 10^−5^	0	0.448	0.01
B	TREX1	c.520A>G	p.R174G	rs759481016	het	NA	LP	-	27	0.17 × 10^−4^	0	0.623	0.02
B	RANBP2	c.8591G>T	p.G2864V	rs765893725	het	NA	VUS	-	28	0.289 × 10^−4^	0	0.999	0
B	TBXAS1	c.319A>G	p.N107D	rs771726219	het	NA	VUS	-	26	0.176 × 10^−4^	0	0.986	0.08

Legend: het = heterozygous; zig = zygosity; seg = segregation; pat = paternal; LP = likely pathogenic; VUS = variant of uncertain significance; MAF = minor allele frequency; NA = not available; hom = homozygous individuals. * expressed considering the ethnic group to whom the patients belong to. ^#^ of homozygous individuals considering all the ethnic groups included in the gnomAD database.

## Data Availability

Any relevant data not evident in this publication, including sequencing data, is available upon request (wayne.hemphill@colorado.edu or fperrino@wakehealth.edu).

## Supplementary Materials

The following supporting information can be downloaded at: https://www.mdpi.com/article/10.3390/genes13071179/s1. Supplemental Figure S1: TREX1 electropherograms of patients A and B. Supplemental Figure S2: Structural context of hTREX1’s mutated residues. Supplemental Figure S3: dsDNA degradation by A136V and R174G variants. Supplemental Figure S4: ssDNA degradation by A136V and R174G variants. Supplemental Figure S5: Quality control data from A136V and R174G preps. Supplemental Table S1: genes responsible for Mendelian conditions causing monogenic forms of stroke.
