# Supplementary material for: Genotype-Phenotype Correlation and Functional Insights for Two Monoallelic TREX1 Missense Variants Affecting the Catalytic Core
Source: Genes (Basel). 2022 Jun 30;13(7):1179. doi: 10.3390/genes13071179 (PMC9323106; doi:10.3390/genes13071179)
Supplement: Supplementary file 1 [file genes-13-01179-s001.zip › genes-1786121-supplementary.pdf]

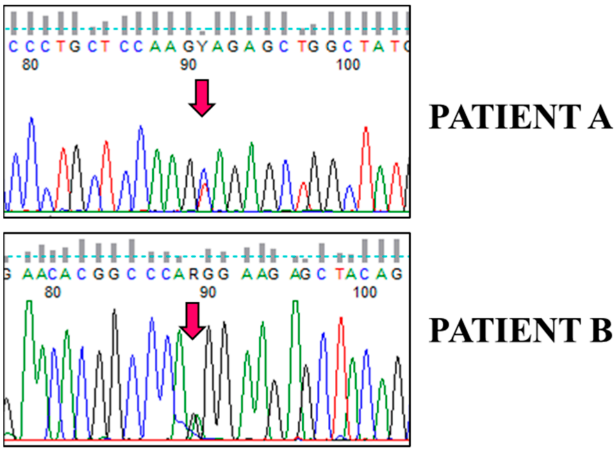

Supplemental Figure S1: **TREX1 electropherograms of patients A and B.** The chromatograms show the presence of c.407C>T (p.A136V) (upper panel) and c.520A>G (p.R174G) (lower panel) variants in patient A and B, respectively.

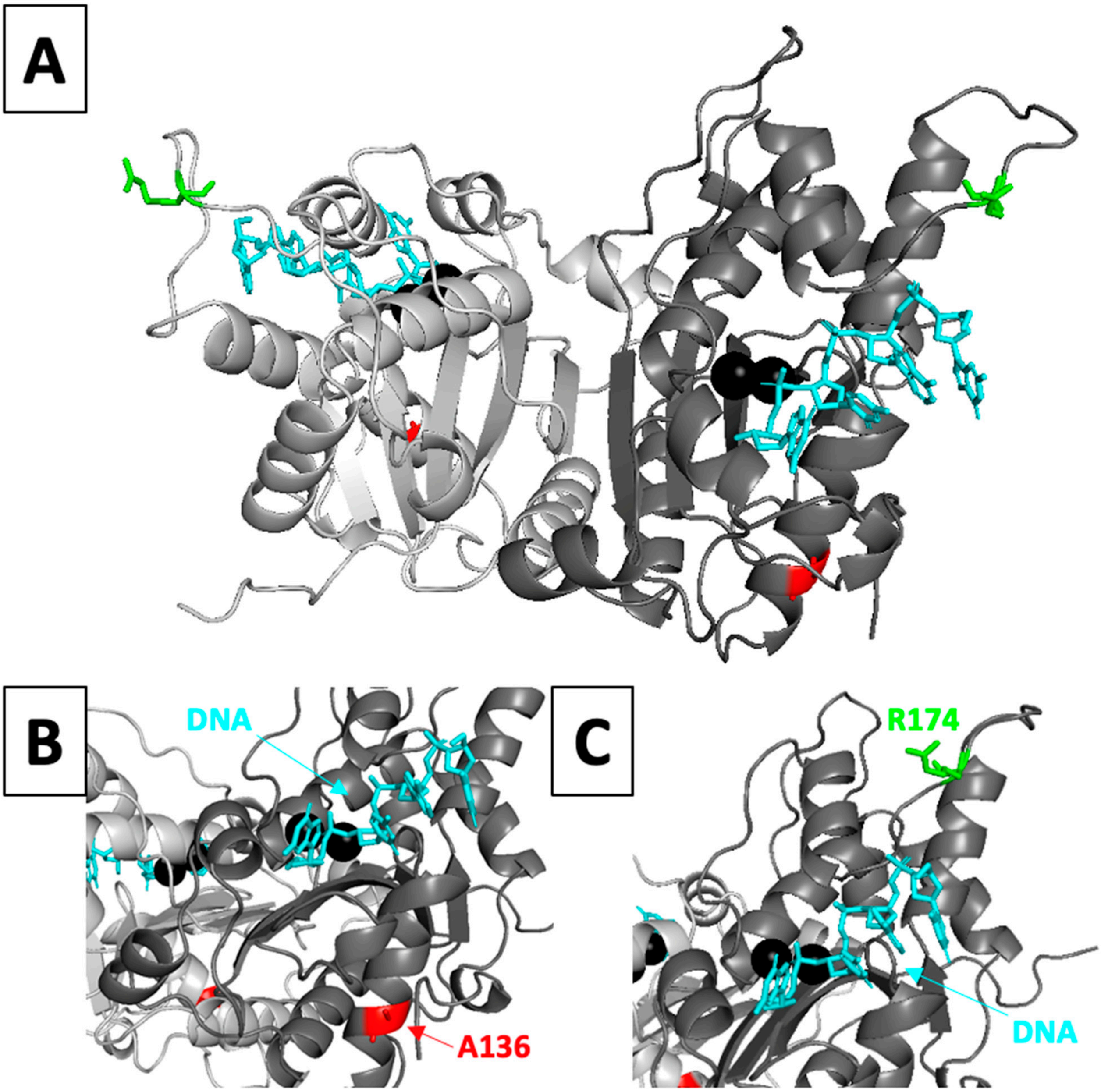

<u>Supplemental Figure S2:</u> Structural context of hTREX1's mutated residues. A structure of mTREX1$_{1\text{-}242}$ bound to ssDNA (PDB = '2IOC') was used as a template with MODELLER v9.22 to model the hTREX1$_{1\text{-}242}$ structure with the originally disordered loops resolved. The respective protomers of the hTREX1 homodimer are shown as dark and light grey cartoons, 4-mer oligos ('DNA') are shown as blue sticks, divalent metal ions are shown as black spheres, A136 residues are shown as red sticks, and R174 residues are shown as green sticks. Graphics were generated with PyMOL v2.3.2 (The PyMOL Molecular Graphics System, Version 2.0 Schrödinger, LLC), and figure was prepared in PowerPoint.

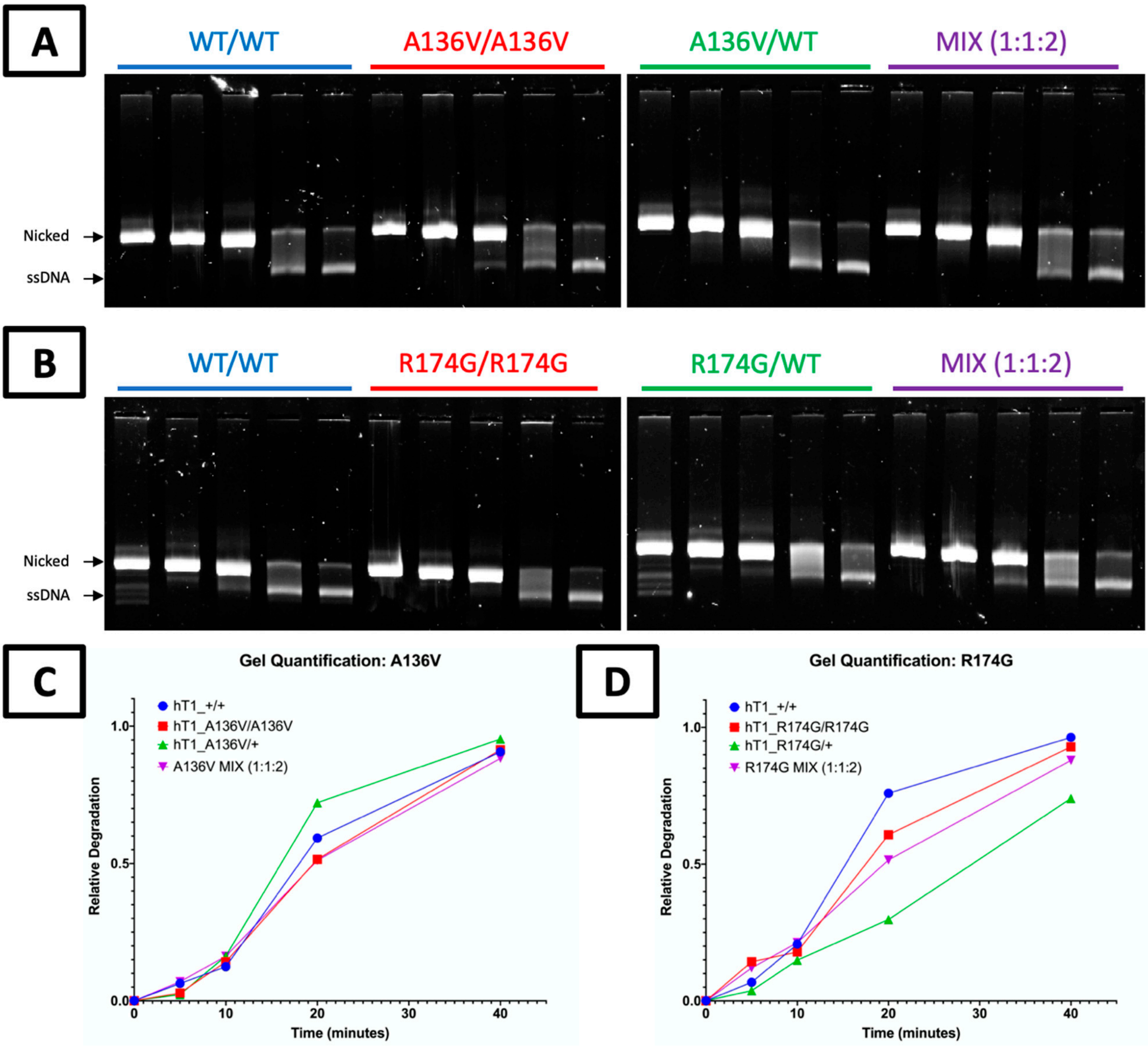

__Supplemental Figure S3__: **dsDNA degradation by A136V and R174G variants. [a,b]** *Agarose Gel Visualization of TREX1 Exonuclease Activity*. Standard exonuclease reactions were prepared with equimolar concentrations of the indicated enzymes, incubated at room temperature for 0, 5, 10, 20, or 40 minutes, quenched in ethanol, then visualized on 0.8% agarose gels. Supercoiled dsDNA plasmid is nicked to generate initial substrate ('Nicked), and TREX1 degrades one strand of the substrate over time to generate ssDNA plasmid ('ssDNA'). **[c,d]** *Quantification of Agarose Gels*. Band intensity for each of the 0-minute lanes on the gels in panels a-b was quantified by densitometry, along with band intensity on the corresponding region of the associated lanes. Differences in band intensity were normalized to the 0-minute lanes' band, and these values ('Relative Degradation') were plotted as a function of time. Gels and plots are representative of two separate experiments.

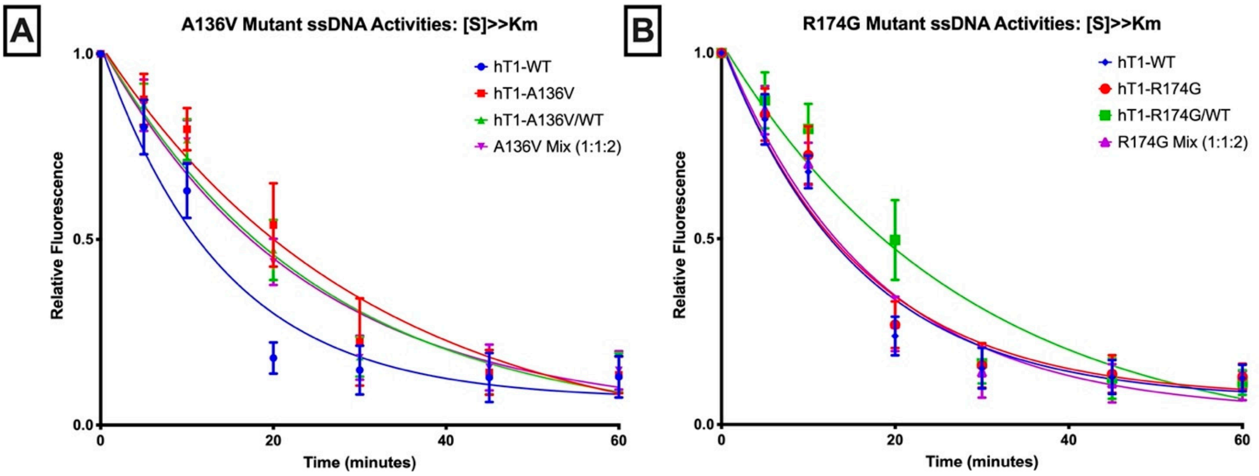

C

Relative Activities

hT1-WT	=	1.0 ± 0.083
hT1-A136V	=	0.58 ± 0.081
hT1-A136V/WT	=	0.67 ± 0.060
A136V MIX (1:1:2)	=	0.71 ± 0.042
hT1-R174G	=	0.87 ± 0.058
hT1-R174G/WT	=	0.64 ± 0.091
R174G MIX (1:1:2)	=	0.89 ± 0.050

Supplemental Figure S4: ssDNA degradation by A136V and R174G variants. [a,b] *Fluorescence-Based Quantification of TREX1 dsDNA Degradation*. Standard exonuclease reactions were prepared with equimolar concentrations of the indicated enzymes, incubated at room temperature for the indicated times, quenched in QuantiFluor ssDNA dye, and ssDNA content measured by fluorescence. Plots of fluorescence vs time were generated and fit with one-phase decay nonlinear regression in Prism 7.0 (GraphPad). Plots are composites of 18 different reactions from 3 different experiments. Data points indicate mean, and error bars represent standard deviation. **[c]** *Activity Rates of TREX1 Variants*. Initial velocities were quantified from the respective regression lines in panels a-b and normalized to wild-type initial velocity to calculate relative activity. Values are mean and standard deviation. '[S] >> K_m' refers to ~5 µM of a 30-mer oligo.

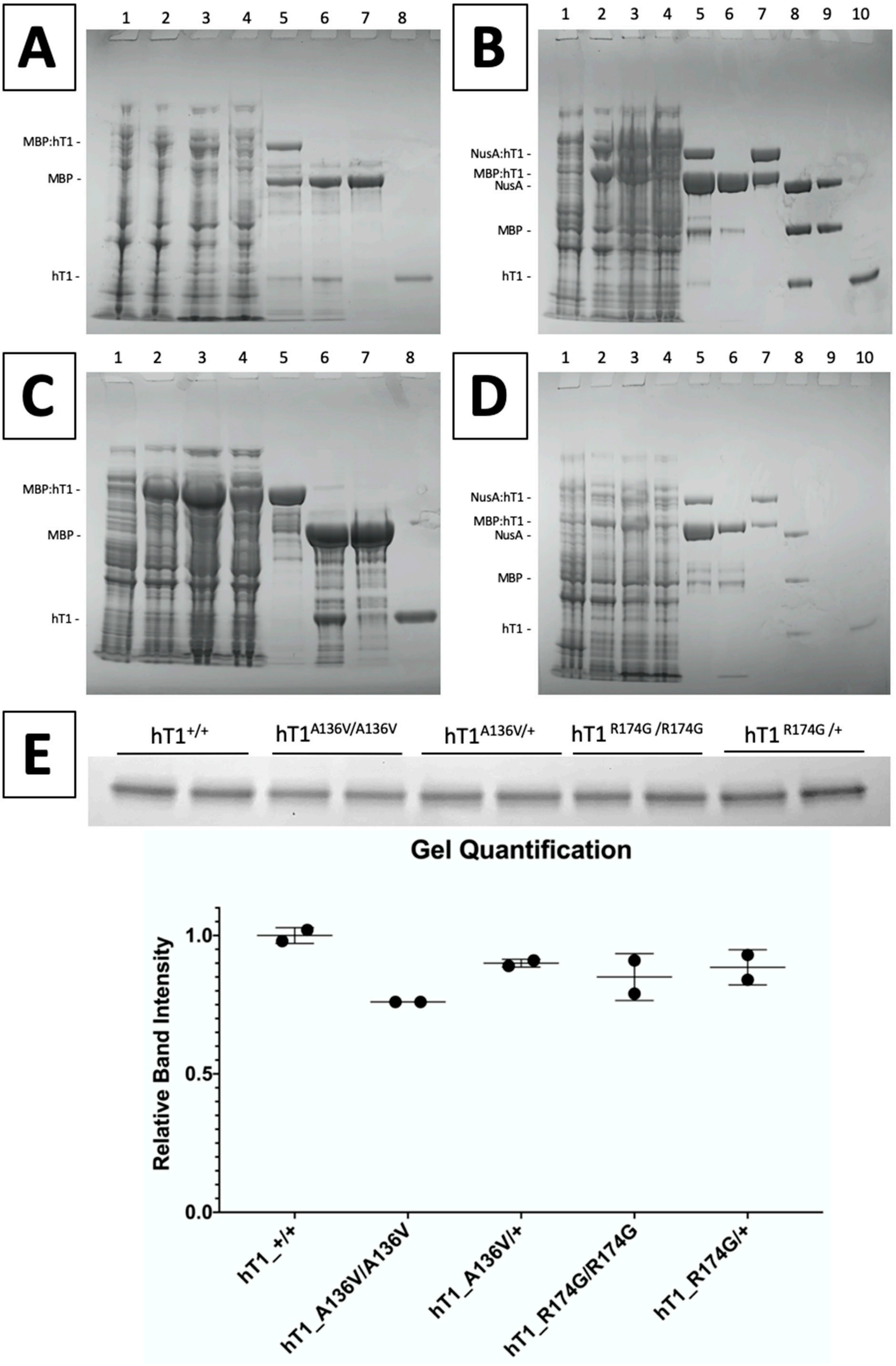
A
1 2 3 4 5 6 7 8
MBP:hT1 -
MBP -
hT1 -
B
1 2 3 4 5 6 7 8 9 10
NusA:hT1 -
MBP:hT1 -
NusA -
MBP -
hT1 -
C
1 2 3 4 5 6 7 8
MBP:hT1 -
MBP -
hT1 -
D
1 2 3 4 5 6 7 8 9 10
NusA:hT1 -
MBP:hT1 -
NusA -
MBP -
hT1 -
E
hT1+/+
hT1A136V/A136V
hT1A136V/+
hT1 R174G /R174G
hT1 R174G /+
Gel Quantification
Relative Band Intensity
1.0
0.5
0.0
hT1_+/+
hT1_A136V/A136V
hT1_A136V/+
hT1_R174G/R174G
hT1_R174G/+

<u>Supplemental Figure S5:</u> Quality control data from A136V and R174G preps. [a,c] *Chromatography Purification of* $hT1^{A136V/A136V}$ *(panel a) &* $hT1^{R174G/R174G}$ *(panel c).* Samples were taken at the indicated steps of chromatography purification and visualized by SDS-PAGE with Coomassie staining. (1) pre-induction culture sample, (2) post-induction culture sample, (3) nickel column applicant, (4) nickel column wash, (5) nickel column eluent, (6) post-cleavage p-cell column applicant, (7) p-cell column wash, & (8) p-cell column eluent. **[b,d]** *Chromatography Purification of* $hT1^{A136V/+}$ *(panel b) &* $hT1^{R174G/+}$ *(panel d).* (1) pre-induction culture sample, (2) post-induction culture sample, (3) amylose column applicant, (4) amylose column wash, (5) amylose column eluent / nickel column applicant, (6) nickel column wash, (7) nickel column eluent, (8) post-cleavage p-cell column applicant, (9) p-cell column wash, & (10) p-cell column eluent. **[e]** *Validation of Enzyme Purities and Concentrations.* Protein content of preps was visualized via SDS-PAGE with Coomassie staining. Enzyme preps were used to load 0.75 µg each, as measured by A_{280}, onto a 12% gel. Band intensities were quantified using GelAnalyzer 19.1, then plotted as mean and range. Graph was prepared with Prism 7.0 (GraphPad), and figure was assembled in PowerPoint.

Supplemental Table S1: genes responsible for Mendelian conditions causing monogenic forms of stroke.

GENE	OMIM # gene	OMIM# disease	inheritance pattern	GenBank ID	coding exons	target submitted (bp)	target designed (bp)	missed (bp)	covered (%)	Reference (PMID)
ABCA1	600046	604091<br>205400	AD<br>AR	NM_005502	49	7276	7276	0	100	30278532
ABCC6	603234	614473<br>264800<br>177850	AR<br>AR<br>AD	NM_001171	31	4903	4755	148	96,98	24008425<br>29709427<br>29722917
ABCD4	603214	614857	AR	NM_005050	19	2771	2771	0	100	20301503
ABCG5	605459	618666	AR	NM_022436	13	2606	2606	0	100	11099417
ABCG8	605460	210250	AR	NM_022437	13	2672	2672	0	100	11099417
ACP5	171640	607944	AR	NM_001111036	4	1178	1178	0	100	26951490
ACTA2	102620	611788<br>613834	AD	NM_001613	8	1214	1214	0	100	19409525<br>30661495
ACTN1	102575	615193	AD	NM_001102	22	3845	3845	0	100	30356112
ACVRL1	601284	600376	AD	NM_000020	9	1602	1602	0	100	31637968
ADAMTS13	604134	274150	AR	NM_139025	29	5734	5722	12	99,79	30356112
ADAR	146920	615010	AR	NM_001111	15	4431	4431	0	100	25672750
ALG8	608103	608104	AR	NM_024079	14	2336	2231	105	95,5	12480927
ANO6	608663	262890	AR	NM_001025356	23	4226	4226	0	100	30356112
APP	104760	605714	AD	NM_201413	19	2545	2545	0	100	7131028

ATP7A	300011	309400	XLR	NM_000052.7	22	4723	4723	0	100	11118799
BGN	301870	300989	XL	NM_001711.6	7	1457	1457	0	100	27632686
BRAF	164757	115150<br>613707<br>613706	AD	NM_001374258.1	18	2481	2481	0	100	12187019
BRCC3	300617	300845	XLR	NM_001018055.3	11	1504	1504	0	100	25733922
C1R	613785	130080	AD	NM_001733.7	9	2336	2336	0	100	25485215
CBS	613381	236800	AR	NM_000071.3	15	1806	1806	0	100	26689889
CCER2	617634	-	-	NM_001243212	5	851	851	0	100	27717682
CCM1	604214	116860	AD	NM_004912	16	2851	2851	0	100	9811928
CCM2	607929	603284	AD	NM_031443.4	11	1868	1868	0	100	9811928
CD59	107271	612300	AR	NM_000611	3	537	537	0	100	25716358
CECR1	607575	182410<br>615688	AR	NM_001282225.2	10	2066	2066	0	100	24552284
COG6	606977	614576	AR	NM_020751.3	20	2996	2996	0	100	20605848
COL1A1	120150	130060	AD	NM_000088.4	51	4905	4905	0	100	28089253<br>12695216
COL1A2	120160	617821<br>225320	AD<br>AR	NM_000089.4	52	4621	4621	0	100	28089253<br>12695216
COL3A1	120180	130050<br>618343	AD<br>AR	NM_000090.4	51	4911	4911	0	100	12786757
COL4A1	120130	611773	AD	NM_001845.6	53	5564	5564	0	100	30413629

		175780<br>618564								
COL4A2	120090	614483	AD	NM_001846.4	47	7489	7489	0	100	30413629
COL4A3	120070	203780<br>104200	AR<br>AD	NM_000091	52	5533	5533	0	100	10893738
COL4A4	120131	203780	AD	NM_000092	47	7423	7423	0	100	10893738
COL4A5	303630	301050	XLD	NM_000495	51	5573	5573	0	100	30045277
COL4A6	303631	-	-	NM_001847	47	7488	7488	0	100	30045277
COL5A1	120215	130000	AD	NM_000093	67	6256	6256	0	100	17053184
COL5A2	120190	130010	AD	NM_000393	54	7200	7200	0	100	17053184
CST3	604312	105150	AD	NM_000099	3	591	591	0	100	3472718
CTSA	613111	-	AD	NM_000308	15	2247	2247	0	100	27664989
CYP11B1	610613	202010<br>103900	AR<br>AD	NM_000497	9	1602	1602	0	100	10852446
DPM1	603503	608799	AR	NM_003859	10	1388	1321	67	95,1	15669674
DYRK1B	604556	615812	AD	NM_004714	10	2390	2390	0	100	24827035
ELN	130160	123700<br>185500	AD	NM_001278939	34	4094	4094	0	100	18348261
ENG	131195	187300	AD	NM_000118	15	2753	2753	0	100	27352867<br>31637968
ENPP1	173335	208000<br>615522	AR<br>AD	NM_006208	25	4028	4028	0	100	19206175

		613312	AR							
EPAS1	603349	611783	AD	NM_001430	16	3413	3413	0	100	18184961
EPHB4	600011	618196	AD	NM_004444	17	3134	3134	0	100	28687708
EPOR	133171	133100	AD	NM_000121	8	1927	1927	0	100	3371213
ESCO2	609353	-	-	NM_001017420	10	2306	2306	0	100	1642282
F10	613872	227600	AR	NM_000504	8	1867	1867	0	100	3732313
F13A1	134570	613225	AR	NM_000129	14	2899	2899	0	100	9550516
F13B	134580	613235	AR	NM_001994	12	2586	2586	0	100	30356112
F2	176930	613679<br>188050	AR<br>AD	NM_000506	14	2009	2009	0	100	28160964
F5	612309	227400<br>188055	AR<br>AD	NM_000130	25	6925	6925	0	100	30635457
F7	613878	227500	AR	NM_000131	9	1761	1761	0	100	30356112
F8	300841	306700	XLR	NM_000132	27	7350	7350	0	100	30356112
F9	300746	306900<br>300807	XLR	NM_000133	8	1466	1466	0	100	19846852
FBLN4	604633	614437	AR	NM_016938	10	1432	1432	0	100	8985490
FBLN5	604580	614434<br>219100<br>608895	AD<br>AR<br>AD	NM_006329	11	1897	1897	0	100	8985490
FBN1	134797	604308<br>154700	AD	NM_000138	65	9266	9266	0	100	29030048

FGA	134820	202400<br>616004	AR<br>AD/AR	NM_021871	6	2705	2705	0	100	19598064
FGB	134830	202400<br>616400	AR<br>AD/AR	NM_005141	8	1876	1876	0	100	19598064
FGG	134850	202400<br>616400	AR<br>AD/AR	NM_000509	10	1877	1877	0	100	19598064
FLI1	193067	617443	AD<br>AR	NM_001271010	10	1491	1491	0	100	30356112
FLNA	300017	300049<br>314400	XLD<br>XL	NM_001110556	47	10294	10294	0	100	9883725
FOXC1	601090	602482	AD	NM_001453	1	1672	1672	0	100	25250569
GAA	606800	232300	AR	NM_001079803	19	3049	3049	0	100	3322184
GCDH	608801	231670	AR	NM_000159	12	1481	1484	0	100	28411331
GDF2	605120	615506	AD	NM_016204	2	1390	1390	0	100	31637968
GFI1B	604383	187900	AD<br>AR	NM_004188	6	1293	1293	0	100	30356112
GGCX	137167	610842<br>277450	digenic<br>AR	NM_000821	15	3053	3053	0	100	12384421
GLA	300644	301500	XL	NM_000169	7	1360	1360	0	100	33835733
GP1BA	606672	231200<br>153670<br>177820	AR<br>AD<br>AD	NM_000173	1	2009	2009	0	100	30356112
GP1BB	138720	231200	AR	NM_000407	2	641	641	0	100	30356112
GP6	605546	614201	AR	NM_016363	8	2263	2263	0	100	30356112

GP9	173515	231200	AR	NM_000174	1	584	584	0	100	30356112
GUCY1A3	139396	615750	AR	NM_000856	8	2157	2157	0	100	25733922
HBB	141900	603903	AR	NM_000518	3	474	474	0	100	7782612
HRG	142640	613116	AD	NM_000412	7	1928	1928	0	100	29108964<br>8475479
HTRA1	602194	600142<br>616779	AR<br>AD	NM_002775	9	1893	1893	0	100	32719647
IFIH1	606951	615846	AD	NM_022168	16	3878	3878	0	100	175395
ITGA2	192974	-	-	NM_002203	30	5046	5046	0	100	30356112
ITGA2B	607759	187800	AD	NM_000419	30	4620	4620	0	100	30356112
ITGB3	173470	619271	AD	NM_000212	15	2517	2517	0	100	30356112
ITM2B	603904	176500<br>117300	AD	NM_021999	6	1101	1101	0	100	10391242
IVD	607036	243500	AR	NM_002225	12	1401	1401	0	100	20142522
JAG1	601920	118450	AD	NM_000214	26	3917	3917	0	100	14993126
JAK2	147796	614521	AD	NM_004972	23	3629	3629	0	100	22397670
JAM3	606871	613730	AR	NM_032801	9	1383	1383	0	100	21109224
KNG1	612358	228960	AR	NM_000893	11	2566	2566	0	100	12576314
KRAS	190070	609942	AD	NM_004985	5	737	737	0	100	12187019
LDLR	606945	143890	AD/AR	NM_001195799	18	2763	2763	0	100	1301956

LDLRAP1	605747	603813	AR	NM_015627	9	1377	1377	0	100	9626156
LMAN1	601567	227300	AR	NM_005570	13	2183	2183	0	100	30356112
LMBRD1	612625	277380	AR	NM_018368	16	2423	2423	0	100	20301503
LMNA	150330	115200	AD	NM_170707	15	2369	2369	0	100	19095983
LOX	153455	617168	AD	NM_001178102	7	1604	1604	0	100	27432961
LZTR1	600574	616564<br>605275	AD<br>AR	NM_006767	21	2733	2733	0	100	12187019
MCFD2	607788	613625	AR	NM_001171507	4	733	733	0	100	30356112
MFAP5	601103	616166	AD	NM_003480	9	972	972	0	100	25434006
MGAT2	602616	212066	AR	NM_002408	1	1394	1394	0	100	19419693<br>1159665
MMACHC	609831	277400	AR	NM_015506	4	889	889	0	100	18986243
MMADHC	611935	277410	AR	NM_015702	7	961	961	0	100	20301409
MPI	154550	602579	AR	NM_002435	8	1672	1672	0	100	10484808
MPL	159530	601977<br>604498	AD<br>AR	NM_005373	12	2028	2028	0	100	15269348
MTHFR	607093	236250	AR	NM_005957	11	2081	2081	0	100	26839351<br>29849257
MTRR	602568	236270	AR	NM_002454	15	2328	2279	49	97,9	29849257
MTR	156570	250940	AR	NM_000254	33	4128	4128	0	100	29849257
MYH11	160745	132900	AD	NM_002474	42	6391	6391	0	100	30661495

MYLK	600922	613780	AD	NM_053025	31	6055	6055	0	100	29544503<br>26147384
NBEAL2	614169	139090	AR	NM_015175	54	10965	10965	0	100	30356112
NF1	613113	162200<br>601321	AD	NM_001042492	58	9161	9161	0	100	30661495
NOTCH1	190198	616028<br>109730	AD	NM_017617	34	8008	7987	21	99,7	25132448<br>9631276
NOTCH3	600276	125310	AD	NM_000435	33	7296	7296	0	100	19539236
NRAS	164790	613224	AD	NM_002524	4	610	610	0	100	12187019
P2RY12	600515	609821	AR	NM_022788	1	1079	1079	0	100	30356112
PCCA	232000	606054	AR	NM_000282	24	2427	2427	0	100	18986243
PCCB	232050	606054	AR	NM_000532	16	2480	2480	0	100	18986243
PCNT	605925	210720	AR	NM_006031	47	12361	12361	0	100	34016138
PDCD10	609118	603285	AD	NM_007217	7	989	989	0	100	17041941
PDE3A	123805	112410	AD	NM_000921	17	4321	4293	28	99,3	25961942
PDE4D	600129	614613	AD	NM_001104631	22	4450	4450	0	100	22464250
PGM1	171900	614921	AR	NM_002633	12	2589	2589	0	100	10484808
PITX2	601542	180500	AD	NM_153426	5	1209	1209	0	100	25250569
PKD1	601313	173900	AD	NM_001009944	46	13372	13278	94	99,3	8130364<br>27567292

PKD2	173910	613095	AD	NM_000297	15	3057	3057	0	100	27567292
PLAU	191840	601709	AD	NM_002658	11	1880	1880	0	100	30356112
PLG	173350	217090	AR	NM_000301	19	2627	2627	0	100	8392398
PLOD1	153454	225400	AR	NM_000302	20	3325	3325	0	100	9617436
PLOD3	603066	612394	AR	NM_001084	19	3167	3167	0	100	18834968
PMM2	601785	212065	AR	NM_000303	8	1141	1141	0	100	29470411
POLR3F	617455	-	AD	NM_006466	9	1041	1041	0	100	30115567
PRDX1	176763	277400	AR	NM_181697	5	650	650	0	100	29302025
PRKACG	176893	616176	AR	NM_002732	1	1066	1066	0	100	30356112
PRKG1	176894	615436	AD	NM_001098512	19	3087	3087	0	100	23910461
PROC	612283	176860<br>612304	AD<br>AR	NM_000312	8	1466	1466	0	100	20187890<br>1511989
PROCR	600646	-	AD	NM_006404	4	757	757	0	100	11552992
PROS1	176880	612336<br>614514	AD<br>AR	NM_000313	16	2287	2287	0	100	20484936
PTPN11	176876	163950	AD	NM_002834	15	1936	1936	0	100	12187019
RAF1	164760	611553	AD	NM_002880	16	2107	2107	0	100	12187019
RANBP2	602752	-	-	NM_006267	29	9965	8963	1002	89,9	29593631
RASA1	139150	608354	AD	NM_002890	26	3412	3411	1	99,9	24038909
RASGRP2	605577	615888	AR	NM_001098671	15	2580	2580	0	100	30356112

RIT1	609591	615355	AD	NM_001256821	6	771	771	0	100	12187019
RNASEH2A	606034	610333	AR	NM_006397	8	1300	1300	0	100	32642802
RNASEH2B	610326	610181	AR	NM_024570	12	1572	1572	0	100	32642802
RNASEH2C	610330	610329	AR	NM_032193	4	695	695	0	100	32642802
RNF213	613768	607151	AD<br>AR	NM_001256071	67	16462	16439	23	99,8	31650369<br>26147384
SAG	181031	258100	AR	NM_000541	15	1968	1968	0	100	22665972
SAMHD1	606754	612952	AR	NM_015474	16	2681	2681	0	100	20842748
SCN5A	600163	614022	AD	NM_001099404	28	6423	6423	0	100	16684018
SERPINC1	107300	613118	AD<br>AR	NM_000488	7	1465	1465	0	100	14347873
SERPIND1	142360	612356	AD	NM_000185	4	1540	1540	0	100	14347873
SERPINE1	173360	613329	AD<br>AR	NM_000602	8	1609	1609	0	100	1435917
SKI	164780	182212	AD	NM_003036	7	2257	2217	40	98,2	24357594
SLC19A2	603941	249270	AR	NM_006996	6	1794	1794	0	100	10720020
SLC20A2	158378	213600	AD	NM_006749	10	2459	2459	0	100	30637044
SLC2A10	606145	208050	AR	NM_030777	5	1876	1876	0	100	17485657
SLFN14	614958	616913	AD	NM_001129820	4	2779	2779	0	100	30356112
SMAD3	603109	613795	AD	NM_005902	10	1452	1452	0	100	21778426

SMAD4	600993	175050	AD	NM_005359	11	1769	1769	0	100	31637968
SMARCAL1	606622	242900	AR	NM_014140	16	3665	3665	0	100	16840568
SOS1	182530	610733	AD	NM_005633	23	4232	4232	0	100	12187019
SOS2	601247	616559	AD	NM_006939	23	4919	4919	0	100	12187019
SPARC	182120	616507	AR	NM_003118	9	1478	1478	0	100	26027498
STAT1	600555	614162	AD	NM_007315	23	2487	2487	0	100	28161409
TBXA2R	188070	614009	AD	NM_001060	3	1423	1245	178	87,5	30356112
TBXAS1	274180	614158	AD	NM_001130966	14	2472	2472	0	100	30356112
TEK	600221	600195	AD	NM_000459	23	4525	4525	0	100	19888299
TGFB2	190220	614816	AD	NM_003238	8	1409	1409	0	100	25835445
TGFB3	190230	615582	AD	NM_003239	7	1589	1589	0	100	25835445
TGFBR1	190181	609192	AD	NM_004612	9	1614	1614	0	100	15731757
TGFBR2	190182	600168	AD	NM_003242	8	1859	1859	0	100	22772368
THBD	188040	614486	AD	NM_000361	1	1738	1738	0	100	26354877
THPO	600044	187950	AD	NM_000460	6	1782	1782	0	100	9425899
TREX1	606609	225750<br>192315	AD/AR<br>AD	NM_033629	1	1160	1160	0	100	11438888
VHL	608537	263400	AR	NM_000551	3	672	672	0	100	14726398
VKORC1	608547	607473	AR	NM_024006	4	776	776	0	100	30356112

VWF	613160	193400<br>277480<br>613554	AD<br>AR<br>AD/AR	NM_000552.5	51	8952	08952	0	100	30356112
XYLT1	608124	264800	AR	NM_022166.4	12	3480	3477	3	99,9	16571645
XYLT2	608125	264800	AR	NM_022167.4	11	3148	3148	0	100	16571645
YY1AP1	607860	602531	AR	NM_001198903	11	2844	2844	0	100	27939641

Legend: AD=autosomal dominant; AR=autosomal recessive; XL=X-linked; bp=base pairs

REFERENCES

1. Perrino, F. W., Miller, H. & Ealey, K. A. Identification of a 3'-->5'-exonuclease that removes cytosine arabinoside monophosphate from 3' termini of DNA. *J. Biol. Chem.* **269**, 16357–16363 (1994).
2. Mazur, D. J. & Perrino, F. W. Excision of 3′ Termini by the Trex1 and TREX2 3′→5′ Exonucleases CHARACTERIZATION OF THE RECOMBINANT PROTEINS. *J. Biol. Chem.* **276**, 17022–17029 (2001).
3. Belyakova, N. V. *et al.* Proof-reading 3'-->5' exonucleases isolated from rat liver nuclei. *Eur. J. Biochem.* **217**, 493–500 (1993).
4. Mazur, D. J. & Perrino, F. W. Identification and Expression of the TREX1 and TREX2 cDNA Sequences Encoding Mammalian 3′→5′ Exonucleases. *J. Biol. Chem.* **274**, 19655–19660 (1999).
5. Silva, U. de *et al.* The Crystal Structure of TREX1 Explains the 3′ Nucleotide Specificity and Reveals a Polyproline II Helix for Protein Partnering. *J. Biol. Chem.* **282**, 10537–10543 (2007).
6. Orebaugh, C. D. *et al.* The TREX1 C-terminal Region Controls Cellular Localization through Ubiquitination. *J. Biol. Chem.* **288**, 28881–28892 (2013).
7. Hasan, M. *et al.* Cytosolic nuclease TREX1 regulates oligosaccharyltransferase activity independent of nuclease activity to suppress immune activation. *Immunity* **43**, 463–474 (2015).
8. Kucej, M., Fermaintt, C. S., Yang, K., Irizarry-Caro, R. A. & Yan, N. Mitotic Phosphorylation of TREX1 C Terminus Disrupts TREX1 Regulation of the Oligosaccharyltransferase Complex. *Cell Rep.* **18**, 2600–2607 (2017).
9. Chowdhury, D. *et al.* The Exonuclease TREX1 Is in the SET Complex and Acts in Concert with NM23-H1 to Degrade DNA during Granzyme A-Mediated Cell Death. *Mol. Cell* **23**, 133–142 (2006).
10. Morita, M. *et al.* Gene-Targeted Mice Lacking the Trex1 (DNase III) 3′→5′ DNA Exonuclease Develop Inflammatory Myocarditis. *Mol. Cell. Biol.* **24**, 6719–6727 (2004).
11. Simpson, S. R., Hemphill, W. O., Hudson, T. & Perrino, F. W. TREX1 – Apex predator of cytosolic DNA metabolism. *DNA Repair* 102894 (2020) doi:10.1016/j.dnarep.2020.102894.
12. Ablasser, A. *et al.* TREX1 Deficiency Triggers Cell-Autonomous Immunity in a cGAS-Dependent Manner. *J. Immunol.* **192**, 5993–5997 (2014).

13. Simpson, S. R. *et al.* T Cells Produce IFN-α in the TREX1 D18N Model of Lupus-like Autoimmunity. *J. Immunol.* (2019) doi:10.4049/jimmunol.1900220.
14. Peschke, K. *et al.* Loss of Trex1 in Dendritic Cells Is Sufficient To Trigger Systemic Autoimmunity. *J. Immunol. Baltim. Md 1950* **197**, 2157–2166 (2016).
15. Sun, L., Wu, J., Du, F., Chen, X. & Chen, Z. J. Cyclic GMP-AMP Synthase Is a Cytosolic DNA Sensor That Activates the Type I Interferon Pathway. *Science* **339**, 786–791 (2013).
16. Kranzusch, P. J., Lee, A. S.-Y., Berger, J. M. & Doudna, J. A. Structure of human cGAS reveals a conserved family of second-messenger enzymes in innate immunity. *Cell Rep.* **3**, 1362–1368 (2013).
17. Civril, F. *et al.* Structural mechanism of cytosolic DNA sensing by cGAS. *Nature* **498**, 332–337 (2013).
18. Gao, P. *et al.* Structure-Function Analysis of STING Activation by c[G(2′,5′)pA(3′,5′)p] and Targeting by Antiviral DMXAA. *Cell* **154**, 748–762 (2013).
19. Zhou, W. *et al.* Structure of the human cGAS–DNA complex reveals enhanced control of immune surveillance. *Cell* **174**, 300-311.e11 (2018).
20. Yang, Y.-G., Lindahl, T. & Barnes, D. E. Trex1 Exonuclease Degrades ssDNA to Prevent Chronic Checkpoint Activation and Autoimmune Disease. *Cell* **131**, 873–886 (2007).
21. Stetson, D. B., Ko, J. S., Heidmann, T. & Medzhitov, R. Trex1 prevents cell-intrinsic initiation of autoimmunity. *Cell* **134**, 587–598 (2008).
22. Rego, S. L. *et al.* TREX1 D18N mice fail to process erythroblast DNA resulting in inflammation and dysfunctional erythropoiesis. *Autoimmunity* **51**, 333–344 (2018).
23. Crow, Y. J. Type I interferonopathies: a novel set of inborn errors of immunity. *Ann. N. Y. Acad. Sci.* **1238**, 91–98 (2011).
24. Rice, G. I. *et al.* Assessment of interferon-related biomarkers in Aicardi-Goutières syndrome associated with mutations in TREX1, RNASEH2A, RNASEH2B, RNASEH2C, SAMHD1, and ADAR: a case-control study. *Lancet Neurol.* **12**, 1159–1169 (2013).
25. Rice, G. I., Rodero, M. P. & Crow, Y. J. Human Disease Phenotypes Associated With Mutations in TREX1. *J. Clin. Immunol.* **35**, 235–243 (2015).

26. Orebaugh, C. D., Fye, J. M., Harvey, S., Hollis, T. & Perrino, F. W. The TREX1 Exonuclease R114H Mutation in Aicardi-Goutières Syndrome and Lupus Reveals Dimeric Structure Requirements for DNA Degradation Activity♦. *J. Biol. Chem.* **286**, 40246–40254 (2011).

27. Rice, G. *et al.* Heterozygous Mutations in TREX1 Cause Familial Chilblain Lupus and Dominant Aicardi-Goutières Syndrome. *Am. J. Hum. Genet.* **80**, 811–815 (2007).

28. Lehtinen, D. A., Harvey, S., Mulcahy, M. J., Hollis, T. & Perrino, F. W. The TREX1 Double-stranded DNA Degradation Activity Is Defective in Dominant Mutations Associated with Autoimmune Disease. *J. Biol. Chem.* **283**, 31649–31656 (2008).

29. Fye, J. M., Orebaugh, C. D., Coffin, S. R., Hollis, T. & Perrino, F. W. Dominant Mutations of the TREX1 Exonuclease Gene in Lupus and Aicardi-Goutières Syndrome. *J. Biol. Chem.* **286**, 32373–32382 (2011).

30. Richards, A. *et al.* C-terminal truncations in human 3′-5′ DNA exonuclease TREX1 cause autosomal dominant retinal vasculopathy with cerebral leukodystrophy. *Nat. Genet.* **39**, 1068–1070 (2007).

31. Pelzer, N. *et al.* Heterozygous TREX1 mutations in early-onset cerebrovascular disease. *J. Neurol.* **260**, 2188–2190 (2013).

32. Tan, R. Y. Y. *et al.* How common are single gene mutations as a cause for lacunar stroke? *Neurology* **93**, e2007–e2020 (2019).

33. McGlasson, S. *et al.* Rare variants of the 3'-5' DNA exonuclease TREX1 in early onset small vessel stroke. *Wellcome Open Res.* **2**, 106 (2017).

34. Hemphill, W. O. & Perrino, F. W. Measuring TREX1 and TREX2 exonuclease activities. *Methods Enzymol.* **625**, 109–133 (2019).

35. Benjamin, P. *et al.* Spectrum of Neuroradiologic Findings Associated with Monogenic Interferonopathies. *Am. J. Neuroradiol.* **43**, 2–10 (2022).

36. Lazea, C., Sur, L. & Florea, M. ROHHAD (Rapid-onset Obesity with Hypoventilation, Hypothalamic Dysfunction, Autonomic Dysregulation) Syndrome—What Every Pediatrician Should Know About the Etiopathogenesis, Diagnosis and Treatment: A Review. *Int. J. Gen. Med.* **14**, 319–326 (2021).

37. Wardlaw, J. M. *et al.* Neuroimaging standards for research into small vessel disease and its contribution to ageing and neurodegeneration. *Lancet Neurol.* **12**, 822–838 (2013).

38. Perrino, F. W., Harvey, S., McMillin, S. & Hollis, T. The Human TREX2 3′ → 5′-Exonuclease Structure Suggests a Mechanism for Efficient Nonprocessive DNA Catalysis. *J. Biol. Chem.* **280**, 15212–15218 (2005).

39. Perrino, F. W. *et al.* Cooperative DNA Binding and Communication across the Dimer Interface in the TREX2 3′ → 5′-Exonuclease. *J. Biol. Chem.* **283**, 21441–21452 (2008).

40. Raraigh, K. S. *et al.* Functional Assays Are Essential for Interpretation of Missense Variants Associated with Variable Expressivity. *Am. J. Hum. Genet.* **102**, 1062–1077 (2018).

41. Richards, S. *et al.* Standards and Guidelines for the Interpretation of Sequence Variants: A Joint Consensus Recommendation of the American College of Medical Genetics and Genomics and the Association for Molecular Pathology. *Genet. Med. Off. J. Am. Coll. Med. Genet.* **17**, 405–424 (2015).

42. Matthijs, G. *et al.* Guidelines for diagnostic next-generation sequencing. *Eur. J. Hum. Genet.* **24**, 1515 (2016).

43. Hamosh, A., Scott, A. F., Amberger, J. S., Bocchini, C. A. & McKusick, V. A. Online Mendelian Inheritance in Man (OMIM), a knowledgebase of human genes and genetic disorders. *Nucleic Acids Res.* **33**, D514–D517 (2005).

44. Rentzsch, P., Witten, D., Cooper, G. M., Shendure, J. & Kircher, M. CADD: predicting the deleteriousness of variants throughout the human genome. *Nucleic Acids Res.* **47**, D886–D894 (2019).

45. Adzhubei, I., Jordan, D. M. & Sunyaev, S. R. Predicting Functional Effect of Human Missense Mutations Using PolyPhen-2. *Curr. Protoc. Hum. Genet. Editor. Board Jonathan Haines Al* **0 7**, Unit7.20 (2013).

46. Ng, P. C. & Henikoff, S. SIFT: predicting amino acid changes that affect protein function. *Nucleic Acids Res.* **31**, 3812–3814 (2003).

47. Kopanos, C. *et al.* VarSome: the human genomic variant search engine. *Bioinformatics* **35**, 1978–1980 (2019).

48. Landrum, M. J. *et al.* ClinVar: public archive of interpretations of clinically relevant variants. *Nucleic Acids Res.* **44**, D862–D868 (2016).

49. Rehder, C. *et al.* Next-generation sequencing for constitutional variants in the clinical laboratory, 2021 revision: a technical standard of the American College of Medical Genetics and Genomics (ACMG). *Genet. Med. Off. J. Am. Coll. Med. Genet.* **23**, 1399–1415 (2021).

50. Volpi, S. *et al.* Efficacy and Adverse Events During Janus Kinase Inhibitor Treatment of SAVI Syndrome. *J. Clin. Immunol.* **39**, 476–485 (2019).
